# Pollutant dispersion and nanoparticle dynamics in magnetized bioconvection for sustainable water treatment

**DOI:** 10.1038/s41598-025-08231-8

**Published:** 2025-07-28

**Authors:** Ehab M. Almetwally, Samah M. Mabrouk, Ahmed S. Rashed, Ehsan H. Nasr

**Affiliations:** 1 Department of Mathematics and Statistics, College of Sciences, Imam Mohammad Ibn Saud Islamic University (IMSIU), Riyadh, 11432 Saudi Arabia; 2 Department of Physics and Engineering Mathematics, Faculty of Engineering, Zagazig University, Zagazig, Egypt; 3 Delta Higher Institute for Engineering and Technology, Mansoura, Egypt

**Keywords:** Bioconvection, Nanoparticle transport, Pollutant dynamics, Hybrid nanofluids, Gyrotactic microorganisms, Ferrofluids, Mathematics and computing, Biological physics

## Abstract

Water pollution has rapidly developed with industrialization and urbanization, making it difficult to sustain water treatment. Traditional methods are ineffective in removing nanoscale contaminants such as heavy metals and microplastics. The present work proposes a new MHD bioconvective hybrid nanofluid system with gyrotactic microorganisms acting under a permanent magnetic field to improve pollutant distribution and extraction. A mathematical model is formulated by integrating continuity, momentum, energy, nanoparticle concentration, microbial motility, and reaction-diffusion equations. The ordinary differential equations (ODEs) are obtained from the model by means of similarity transformations. Numerical solutions show that combining bioconvection with magnetic control greatly improves pollutant removal efficiency. Thermophoresis and Brownian motion help move nanoparticles. Increasing the Hartmann number slows fluid velocity due to Lorentz forces. At the same time, a higher bioconvection Péclet number encourages an even distribution of bacteria, which helps with pollutant spread. Validation against existing literature confirms the model’s correctness. This method provides a sustainable and energy-efficient way to purify water, using microbial dynamics and magnetic control for environmental cleanup.

## Introduction

One of the critical issues facing the 21st century is potable water. The high-speed rate of industrialization coupled with agricultural runoff and increased urbanization has led to an unprecedented level of water pollution over the last few years, which conventional methods of water treatment are not sufficient to counter these events. Numerous pollutants, such as nanoparticles, microplastics, and heavy metals, are immensely difficult to remove because of their small size and intricate interactions within the aquatic environments. In the increasing face of this challenge, new, innovative, and sustainable water treatment methodologies that function well at the nanoscale are developed. The behavior of small, motile microorganisms like bacteria and algae are responsible of initializing the bioconvection process. Due to suspension in a fluid environment, these microorganisms are swimming upwards, hence creating convection currents which are responsible for the circulation of fluid. This approach, most likely, will increase the transportation of pollutants and sedimentation processes while providing a non-ecologically destructive and energy-efficient way of purifying water. The combination of bioconvection with magnetized fluids -such as ferrofluids-raises intriguing questions. Magnetized fluids contain very small magnetic particles, which respond in a quite predictable way to the outer magnetic fields. It thus becomes possible to modulate, direct, and enhance the flow characteristics of fluid otherwise unachievable using ordinary fluids. These developments open up promising prospects concerning the enhancement of nanoparticle transport and the remediation of pollutants in aquatic systems^1^.

Bioconvection occurs when motile microorganisms, such as Bacillus subtilis bacteria or algae, swim preferentially towards favorable environments, which are the regions with more oxygen or light. As the microorganisms move upwards in the fluid, they create areas of reduced density, which in turn trigger natural convection currents. The result is a self-organized mixing mechanism that improves the transport of nutrients, particles, and potentially harmful agents through the fluid environment^1^.

Hybrid nanofluids, containing two or more types of nanoparticles, exhibit superior thermal and rheological properties compared to conventional nanofluids, making them ideal for heat transfer applications.

Bioconvection has already been studied in several laboratory studies, where the mixing capacity of this bioconvection was proven effective. At the environmental application scale, this natural convection phenomenon could help attack one major challenge in the field: being able to diffuse pollutants out swiftly and capture them for a successful removal. Jawad et al.^2^ investigated the case of induced magnetic field alongside a horizontal sheet immersed in hybrid nanofluid (HNF) incorporating swimming microorganisms. Shah et al.^3^ provided a bioconvection analysis of a fluid activated externally by an energy source. The results emphasized that the raise in the magnetic field parameter implied a decrease in porosity. In their model, Rashed et al.^4,5^ studied the characteristics of nanofluid surrounding a vertical plate and cylindrical pipes, respectively. Mabrouk et al.^6^ employed their experience to detect the effect of the power index on the hybrid nanofluid inside solar collector. They found that the velocity components are improved as the power-law index rises. The same authors in^7^ numerically simulated the entropy and thermal behavior in solar collectors. They stated that the inclination angle of the solar panel critically affects the collected temperature and the fluid velocity.

Chu et al.^8^ reported that the distribution of microorganisms decreased with an increase in the Peclet number. The authors further contributed that an enhanced velocity profile enhances the wedge angle parameters for static as well as moving wedges. Further, the velocity of the Falkner-Skan nanofluid was found to decrease with the increase in infinite shear rate viscosity, bioconvection Rayleigh number, and buoyancy ratio. The unique manifolds method and Lie infinitesimals have been utilized to obtain solutions to some fluid dynamics problems^9–11^ and evolution Eqs.^12,13^, respectively. Sarma and Paul^14^ analyzed bioconvection in an Ag-CuO/H_2_O Ellis hybrid nanofluid with motile bacteria adjacent to a stretched cylindrical tube. Results indicated that an increase in the Ellis fluid parameter caused velocity to increase while, concurrently, reducing temperature and concentration profiles. Conversely, thermophoresis and Brownian motion were observed to enhance mass transfer rates but with an inverse dependence for slip effects. Furthermore, the Peclet number and bioconvective constants exhibited interesting but opposite trends as compared to gyrotactic microorganism profiles, which were notable findings. Paul et al.^15^ explored mixed convection in a hybrid nanofluid via a radiative cone and found Casson nanofluid significantly improved tangential skin friction by 52%. Thermal behavior of magnetohydrodynamic (MHD) flow with Al_2_O_3_-Cu-TiO_2_/H_2_O was investigated by Rafique et al.^16^, who found that an increase in magnetic parameters led to a decrease in temperature and enhancement in velocity. Also, Paul et al. studied Casson-Maxwell hybrid nanofluids’ unsteady flow with temperature-dependent thermal conductivity^17^ and concluded that Casson-Maxwell hybrid nanofluid resulted from mixing Casson and Maxwell nanofluids led to very good skin friction increase, a 11% enhancement in heat transfer compared to the Casson-Maxwell nanofluid. Mahmood et al.^18^ analyzed tri-hybrid nanofluid flow numerically over a sheet. Islam et al.^19^ studied electroosmotic flow with ternary hybrid nanoparticles and concluded that the thermo-migration factor reduced the mass transport rate, while the Peclet number resulted in a reduction in the density of motile microbes. In addition, Islam et al.^20^ discussed the flow dynamics of Carreau nanomaterial with the influence of a chemical reaction, showing that variations in the Hartmann number increased mobility of nanomaterial and that the variable for radiation created opposing effects on heat transfer rate and entropy generation. Rana et al.^21,22^ investigated entropy-optimized nano-bioconvective flow and microbial transport within blood flow and revealed that blood velocity decreased with the increase in the Williamson factor and increased with the viscosity ratio. Islam et al.^23^ conducted a numerical analysis of MHD flow and reported that increased Rayleigh numbers and increased nanoparticle concentrations enhanced the thermal performance of hybrid nanofluids, while effects of the magnetic field were the reverse. Nanofluids can be categorized into mono, hybrid, and ternary hybrid nanofluids, whose primary purpose is to utilize the properties of multiple types of nanoparticles rather than relying on one specific type. Samrity and Yin^24^ studied the performance of a pulsating heat pipe and revealed that Al_2_O_3_-Cu hybrid nanofluid exhibited 30–54% lower thermal resistance compared to water at the same filling ratio and heat input, indicating enhanced efficiency in heat transfer. S. E. Bone et al.^25^ investigated a clean water technology’s advanced characterisation. M. T. Van Vliet et al.^26^ examined the quality of surface water and the development of clean water technology in relation to global water shortage. F. Macedonio et al.^27^ investigated effective technologies for the global supply of clean water. A. Nagar and T. Pradeep^28^ examined Nanotechnology for clean water: requirements, gaps, and solutions. N. Acharya^29^ examined how the curved fins affected the hydrothermal changes and entropy analysis of buoyancy-driven MWCNT-Fe3O4-H2O hybrid nanofluid flow inside an annular enclosure. N. Acharya^30^ investigated Interpreting the impact of the curvature of fitted curved fins on the flow patterns and entropy analysis of buoyancy-driven magnetized hybrid nanofluidic transport in an annular enclosure. N. Acharya^31^ examined the impact of nanoparticle diameter and the solid-liquid interfacial layer on the flow patterns and thermal control of radiative nanofluid spraying on an inclined rotating disk using spectral modeling. N. Acharya^32^ investigated the thermal behavior and flow patterns of a hybrid nanofluid flow within a microchannel when radiative solar radiation is present. N. Acharya^33^ studied spectral quasi linearization modeling of hybrid nanofluid spraying on an inclined spinning disk to determine its hydrothermal behavior. R. Adhikari and S. Das^34^ examined the entropy assessment of microbial dynamics in a Casson-Maxwell-Oldroyd-B nanofluid on a tilted elongated cylinder utilizing a reactive magnet. S. Sarkar and S. Das^35^ examined the dynamics of a cross nanofluid containing oxytactic microorganisms around a stretchable cylinder under the influence of nonlinear thermal radiation, Lorentz force, and Arrhenius activation energy. R. Adhikari and S. Das^36^ examined biological transmission in an entropy framework using a magnetized reactive Casson-Maxwell nanofluid across a slanted stretchable cylinder. S. Sarkar and S. Das^37^ examined gyrotactic microorganisms with Arrhenius kinetics swimming in magneto-Sutterby nanofluid across a sliding cylinder in a Darcy-Forchheimer porous space. S. Sarkar and S. Das^38^ examined how a stretchy cylindrical surface placed in a DF porous media caused gyrotactic microorganisms to travel in a magneto-nano-polymer susceptible to Arrhenius kinetics and non-linear radiation. A. Ali, S. Sarkar and S. Das^39^ examined oxytactic microorganisms being transported along a flexible cylinder using bioconvective chemically reactive entropy optimized cross-nanomaterial with Lorentz force and Arrhenius kinetics. I. L. Animasaun et al.^40^ investigated Evaluation, introduction, and meta-analysis of the momentum diffusivity to thermal diffusivity ratio. F. Wang et al.^41^ examined dynamics across three t-shaped duct inlets: Inlet velocity’s impact on the turbulence-prone transient air and water encountering cold fronts. I. Animasaun et al.^42^ investigated the effect of turbulent water dynamics on the half-cycle length of a convergent circular wavy duct with a diverging outlet. H. Zhu et al.^43^ examined a hybrid system’s performance prediction and control approach based on an annular thermoelectric generator and tubular solid oxide fuel cell.

A review of the existing literature shows that there is no existing research that has explored ternary hybrid nanofluids’ three-dimensional bio-convection flow in an inclined microchannel. The existing study is hence motivated by the need to investigate three-dimensional flow characteristics under the combined effects of a solar radiation and a magnetic field.

Ferrofluids, which are also known as magnetized fluids, provide one solution to this challenge. Composed of magnetic nanoparticles, these fluids can be manipulated with exterior magnetic fields, allowing for control with considerable precision. Under a magnetic field, ferrofluids can, for example, be made to flow in specified directions, either to agglomerate the particles or capture contaminants in the fluid. These qualities have been leveraged with great success in applications spanning thermal management systems to medical devices. In the context of water treatment, magnetized fluids could give the control lacking in bioconvection. Magnetic fields will be applied to take control of the way pollutants and nanoparticles move around in the fluid, directing them to zones where they can be removed or annihilated. Combined with the natural mixing provided by bioconvection, it is due to the controllability of magnetized fluids that water purification technologies could find a new milestone. The mixing of fluid is done by the microorganisms, and this, assisted by the magnetic field, works to control the flow of the fluid and movement of particles. This synergy enhances the movement of pollutants and thus opens new ways to deal with tiny contaminants that normal methods cannot handle. Such synergy makes the system even better than the sum of its parts when bioconvection and magnetized fluids come together. But whereas scientists have looked at each part of this system bioconvection and magnetized fluids-together, their combined effects are still mostly unknown. How do tiny living things act in a magnetized fluid? How does a magnetic field affect how nanoparticles move and group together with bioconvection? These are the questions we want to answer^1^.

This work examines the behavior of magnetized nanofluids containing motile microorganisms that induce bioconvection, with particular emphasis on the transport of nanoparticles and models the dispersion of pollutants for environmental assessment. Magnetic nanofluids have been studied for improving the heat transfer and pollution decomposition in a transient magnetic field. This study uniquely considers bio-convective hybrid nanofluids under a steady external magnetic field, expanding prior MHD models. Absence of unified models taking into account thermophoresis, Brownian motion, magnetic field effect, and gyrotactic nature. As to the best of our knowledge, no effort to investigate the simultaneous influence of magnetic fields and bacteria dynamics on pollutant removal efficiency is made possible in the literature.

We need to create a complete Model by combine the dynamics of hybrid nanoparticles, gyrotactic behavior, and persistent magnetic field effects into a single mathematical framework. Expand to 3D Flow Analysis to depict intricate fluid dynamics in constrained geometries and microchannels, develop more lifelike, three-dimensional models. Parametric research to comprehend the relationships between physical characteristics and how they affect flow stability and pollution dispersion, do thorough parametric studies. Validation in Practice by use tests and simulations to validate the model, paying particular attention to practical uses such as biomedical devices and water treatment.

By combining thermophoresis, Brownian motion, magnetic effects, and gyrotactic behavior into a single model, the work is novel and offers a more realistic depiction of industrial and natural systems. Optimizing the removal of pollutants and the transfer of nutrients in fluid systems is made possible by enhanced pollutant removal. Moreover, deep parametric analysis is introduced.

## Mathematical formulation

**Fig. 1:**
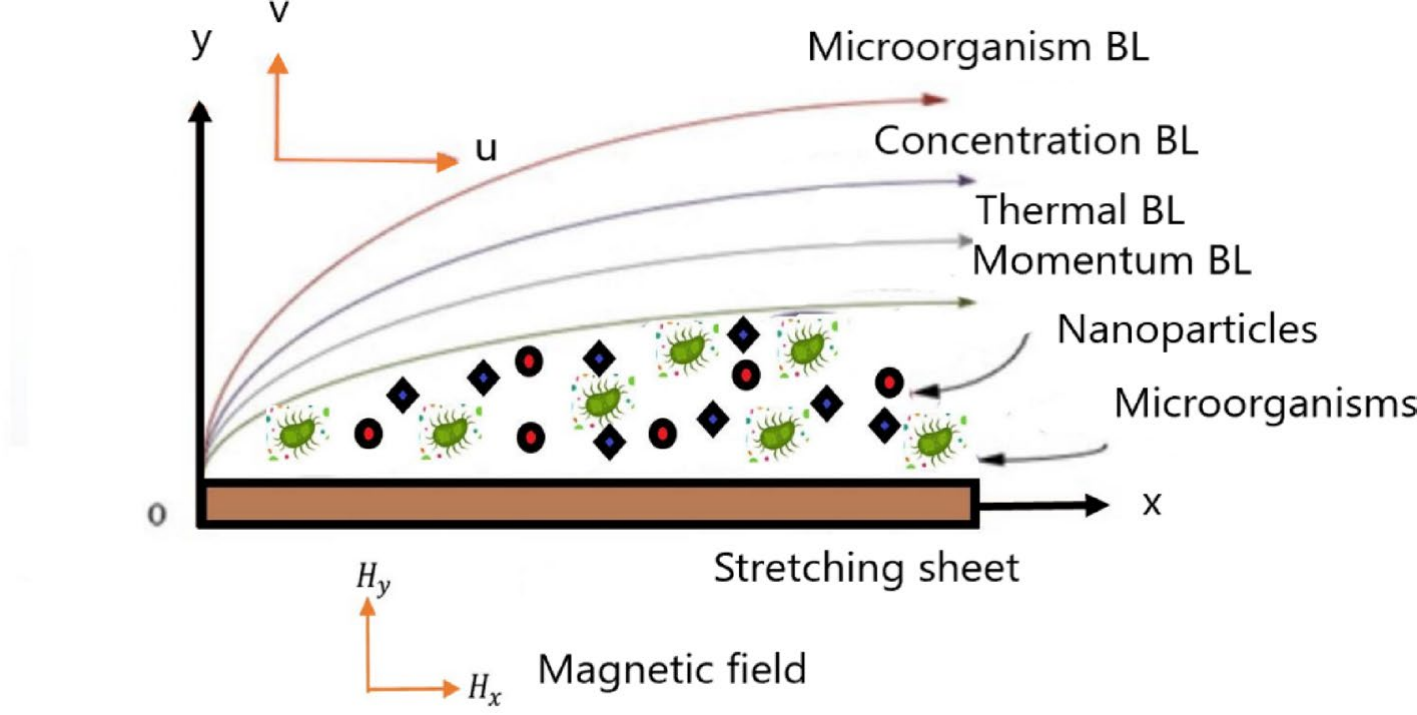
Physical representation of the problem.

In the present study, the magnetohydrodynamic (MHD) flow over a stretching sheet is investigated. The base fluid is water, while the nanoparticles consist of a hybrid nanofluid composed of non-magnetic Cu and CuO particles in a magnetized base fluid. Gyrotactic microorganisms and pollutants are dispersed throughout the fluid domain and exhibit upward swimming behavior. Additionally, the flow dynamics are influenced by an external permanent magnetic field. The mathematical model of the current problem includes continuity Eq. (1), momentum Eq. (2), energy Eq. (3), nanoparticles concentration Eq. (4), microorganism continuity Eq. (5), Maxwell’s magnetic field Eqs. (6) and (7), a reaction-diffusion Eq. (8) for reacting species and a pollutant dispersion Eq. (9) for pollutant transport and decay. As illustrated in Fig. 1, which depicts the precise configuration of the system, the implications of this study extend to biotechnological applications and environmental management. The mathematical model is created using Navier-Stokes, reaction-diffusion, and pollutant dispersion Eqs.^1,4,8,44–49^.

1 $$\:{u}_{x}+{v}_{y}=0$$

2 $$\begin{gathered} \:u_{t} + uu_{x} + vu_{y} = \frac{{\mu \:_{{hnf}} }}{{\rho \:_{{hnf}} }}\left( {\frac{{\partial \:}}{{\partial \:y}}\left( {\frac{{\partial \:u}}{{\partial \:y}}} \right)} \right) - \frac{{\sigma \:B_{0}^{2} u}}{{\rho \:_{{hnf}} }} + \beta \:g_{1} \left( {T - T_{{\infty \:}} } \right) \hfill \\ - \frac{{\rho \:_{p} - \rho \:_{f} }}{{\rho \:_{{hnf}} }}g_{1} \left( {C - C_{{\infty \:}} } \right) - \frac{{\rho \:_{m} - \rho \:_{f} }}{{\rho \:_{{hnf}} }}g_{1} \left( {N - N_{{\infty \:}} } \right) + \frac{{\mu \:_{e} H_{2} }}{{4\pi \:\rho \:_{{hnf}} }}\left( {H_{{1x}} + H_{{1y}} } \right) \hfill \\ \end{gathered}$$

3 $$\:{T}_{t}+u{T}_{x}+v{T}_{y}=\frac{{K}_{hnf}}{{\left(\rho\:{c}_{p}\right)}_{hnf}}\frac{{\partial\:}^{2}T}{\partial\:{y}^{2}}+\left[{D}_{B}\frac{\partial\:C}{\partial\:y}\frac{\partial\:T}{\partial\:y}+\frac{{D}_{T}}{{T}_{\infty\:}}{\left(\frac{\partial\:T}{\partial\:y}\right)}^{2}\right]+\frac{Q}{{\left(\rho\:{c}_{p}\right)}_{hnf}}\left(T-{T}_{\infty\:}\right)$$

4 $$\:{C}_{t}+u{C}_{x}+v{C}_{y}={D}_{B}{(C}_{xx}+{C}_{yy})$$

5 $$\:{N}_{t}+u{N}_{x}+v{N}_{y}+\frac{b{w}_{c}}{{C}_{w}-{C}_{\infty\:}}\left[\frac{\partial\:N}{\partial\:y}\right]={D}_{n}{(N}_{xx}+{N}_{yy})$$

6 $$\:{{H}_{1}}_{t}+u{{H}_{1}}_{x}+v{{H}_{1}}_{y}={\eta\:}_{0}\left(\frac{{\partial\:}^{2}{H}_{1}}{\partial\:{y}^{2}}\right)$$

7 $$\:{{H}_{2}}_{t}+u{{H}_{2}}_{x}+v{{H}_{2}}_{y}={\eta\:}_{0}\left(\frac{{\partial\:}^{2}{H}_{2}}{\partial\:{y}^{2}}\right)$$

8 $$\:{R}_{t}+u{R}_{x}+v{R}_{y}={D}_{r}{(R}_{xx}+{R}_{yy})-{k}_{r}({c}_{1}^{*}{H}_{1}-{c}_{2}^{*}{H}_{2})$$

9 $$\:{P}_{t}+u{P}_{x}+v{P}_{y}={D}_{p}{(P}_{xx}+{P}_{yy})-\alpha\:P+\gamma\:\left(T-{T}_{\infty\:}\right)+{\zeta\:}^{*}\left(C-{C}_{\infty\:}\right)+\delta\:({H}_{1}^{2}+{H}_{2}^{2})$$

Boundary condition:

$$\:y=0$$

$$\:u={u}_{w}(x,t),\:{H}_{1}={H}_{1w}(x,t),\:{H}_{2}={H}_{2w}(x,t),\:R={R}_{w}(x,t),P={P}_{w}(x,t),T={T}_{w},\:\:C={C}_{w},\:\:N={N}_{w}$$

$$\:y\to\:\infty\:$$

10 $$\:u=0,\:{H}_{1}=0,\:{H}_{2}=0,R=0,P=0,\:T={T}_{\infty\:},\:\:C={C}_{\infty\:},\:\:N={N}_{\infty\:}$$

Normalization process:

$$\:u\left(x,y,t\right)={u}^{*}\left(x,y,t\right){u}_{w}\left(x,t\right)$$

$$\:{H}_{1}\left(x,y,t\right)={H}_{1}^{*}\left(x,y,t\right){{H}_{1}}_{w}\left(x,t\right)$$

$$\:{H}_{2}\left(x,y,t\right)={H}_{2}^{*}\left(x,y,t\right){{H}_{2}}_{w}\left(x,t\right)$$

$$\:R\left(x,y,t\right)={R}^{*}\left(x,y,t\right){R}_{w}\left(x,t\right)$$

$$\:P\left(x,y,t\right)={P}^{*}\left(x,y,t\right){P}_{w}\left(x,t\right)$$

11 $$\:T=\varDelta\:T\theta\:+{T}_{\infty\:}\:\:\:\:\:\:\:\:\:\:\:C=\varDelta\:C{C}^{*}+{c}_{\infty\:}\:\:\:\:\:\:\:\:\:\:\:\:\:\:N=\varDelta\:N{N}^{*}+{N}_{\infty\:}$$

The following relations are employed to simplify the model:

12 $$\begin{gathered} \:a_{1} = \frac{{\mu \:_{{hnf}} }}{{\rho \:_{{hnf}} }},a_{2} = \frac{{\sigma \:B_{0}^{2} }}{{\rho \:_{{hnf}} }},a_{3} = \beta \:g_{1} ,a_{4} = \frac{{\rho \:_{p} - \rho \:_{f} }}{{\rho \:_{{hnf}} }}g_{1} , \hfill \\ a_{5} = \frac{{\rho \:_{m} - \rho \:_{f} }}{{\rho \:_{{hnf}} }}g_{1} ,a_{6} = \frac{{\mu \:_{e} }}{{4\pi \:\rho \:_{{hnf}} }},a_{7} = \frac{{K_{{hnf}} }}{{\left( {\rho \:c_{p} } \right)_{{hnf}} }},a_{8} = D_{B} , \hfill \\ a_{9} = \frac{{D_{T} }}{{T_{{\infty \:}} }},a_{{10}} = \frac{Q}{{\left( {\rho \:c_{p} } \right)_{{hnf}} }},a_{{11}} = \frac{{bw_{c} }}{{C_{w} - C_{{\infty \:}} }},a_{{12}} = D_{n} \hfill \\ \end{gathered}$$

Equation (1) through (9) are transformed to:

13 $$\:{u}^{*}\frac{\partial\:{u}_{w}}{\partial\:x}+{u}_{w}\frac{\partial\:{u}^{*}}{\partial\:x}+\frac{\partial\:v}{\partial\:y}=0$$

14 $$\begin{gathered} u^{*} \frac{{\partial u_{w} }}{{\partial t}} + u_{w} \frac{{\partial u^{*} }}{{\partial t}} + u^{*} u_{w} \left( {u^{*} \frac{{\partial u_{w} }}{{\partial x}} + u_{w} \frac{{\partial u^{*} }}{{\partial x}}} \right) + v\left( {u_{w} \frac{{\partial u^{*} }}{{\partial y}}} \right) \hfill \\ = a_{1} \left( {u_{w} \frac{{\partial ^{2} u^{*} }}{{\partial y^{2} }}} \right) - a_{2} u^{*} u_{w} + a_{3} \Delta T\theta - a_{4} \Delta CC^{*} - a_{5} \Delta NN^{*} \hfill \\ + a_{6} H_{2}^{*} H_{{2w}} \left( {H_{1}^{*} \frac{{\partial H_{{1w}} }}{{\partial x}} + H_{{1w}} \frac{{\partial H_{1}^{*} }}{{\partial x}} + H_{{1w}} \frac{{\partial H_{1}^{*} }}{{\partial y}}} \right) \hfill \\ \end{gathered}$$

15 $$\:{\Delta\:}T\frac{\partial\:\theta\:}{\partial\:t}+{u}^{*}{u}_{w}{\Delta\:}T\frac{\partial\:\theta\:}{\partial\:x}+v{\Delta\:}T\frac{\partial\:\theta\:}{\partial\:y}={a}_{7}{\Delta\:}T\frac{{\partial\:}^{2}\theta\:}{\partial\:{y}^{2}}+\left[{a}_{8}{\Delta\:}C\frac{\partial\:{C}^{*}}{\partial\:y}{\Delta\:}T\frac{\partial\:\theta\:}{\partial\:y}+{a}_{9}{\left({\Delta\:}T\frac{\partial\:\theta\:}{\partial\:y}\right)}^{2}\right]+{a}_{10}\varDelta\:T\theta\:$$

16 $$\:\frac{\partial\:{C}^{*}}{\partial\:t}+{u}^{*}{u}_{w}\frac{\partial\:{C}^{*}}{\partial\:x}+v\frac{\partial\:{C}^{*}}{\partial\:y}={D}_{B}\left(\frac{{\partial\:}^{2}{C}^{*}}{\partial\:{x}^{2}}+\frac{{\partial\:}^{2}{C}^{*}}{\partial\:{y}^{2}}\right)$$

17 $$\:\frac{{\partial\:N}^{*}}{\partial\:t}+{u}^{*}{u}_{w}\frac{{\partial\:N}^{*}}{\partial\:x}+v\frac{{\partial\:N}^{*}}{\partial\:y}+{a}_{11}\left[\frac{{\partial\:N}^{*}}{\partial\:y}\right]={a}_{12}\left(\frac{{{\partial\:}^{2}N}^{*}}{\partial\:{x}^{2}}+\frac{{{\partial\:}^{2}N}^{*}}{\partial\:{y}^{2}}\right)$$

18 $$\:{H}_{1}^{*}\frac{{{\partial\:H}_{1}}_{w}}{\partial\:t}+{{H}_{1}}_{w}\frac{{\partial\:H}_{1}^{*}}{\partial\:t}+{u}^{*}{u}_{w}\left({H}_{1}^{*}\frac{{{\partial\:H}_{1}}_{w}}{\partial\:x}+{{H}_{1}}_{w}\frac{{\partial\:H}_{1}^{*}}{\partial\:x}\right)+v{{H}_{1}}_{w}\frac{{\partial\:H}_{1}^{*}}{\partial\:y}={\eta\:}_{0}\left({{H}_{1}}_{w}\frac{{{\partial\:}^{2}H}_{1}^{*}}{\partial\:{y}^{2}}\right)$$

19 $$\:{H}_{2}^{*}\frac{{{\partial\:H}_{2}}_{w}}{\partial\:t}+{{H}_{2}}_{w}\frac{{\partial\:H}_{2}^{*}}{\partial\:t}+{u}^{*}{u}_{w}\left({H}_{2}^{*}\frac{{{\partial\:H}_{2}}_{w}}{\partial\:x}+{{H}_{2}}_{w}\frac{{\partial\:H}_{2}^{*}}{\partial\:x}\right)+v{{H}_{2}}_{w}\frac{{\partial\:H}_{2}^{*}}{\partial\:y}={\eta\:}_{0}\left({{H}_{2}}_{w}\frac{{{\partial\:}^{2}H}_{2}^{*}}{\partial\:{y}^{2}}\right)$$

20 $$\begin{gathered} \:R^{*} \frac{{\partial \:R_{w} }}{{\partial \:t}} + R_{w} \frac{{\partial \:R^{*} }}{{\partial \:t}} + u^{*} u_{w} \left( {R^{*} \frac{{\partial \:R_{w} }}{{\partial \:x}} + R_{w} \frac{{\partial \:R^{*} }}{{\partial \:x}}} \right) + vR_{w} \frac{{\partial \:R^{*} }}{{\partial \:y}} \hfill \\ = D_{r} \left( {R^{*} \frac{{\partial \:^{2} R_{w} }}{{\partial \:x^{2} }} + 2\frac{{\partial \:R_{w} }}{{\partial \:x}}\frac{{\partial \:R^{*} }}{{\partial \:x}} + R_{w} \frac{{\partial \:^{2} R^{*} }}{{\partial \:x^{2} }} + R_{w} \frac{{\partial \:^{2} R^{*} }}{{\partial \:y^{2} }}} \right) \hfill \\ - k_{r} (c_{1}^{*} H_{1}^{*} H_{{1w}} - c_{2}^{*} H_{2}^{*} H_{{2w}} ) \hfill \\ \end{gathered}$$

21 $$\begin{gathered} \:P^{*} \frac{{\partial \:P_{w} }}{{\partial \:t}} + P_{w} \frac{{\partial \:P^{*} }}{{\partial \:t}} + u^{*} u_{w} \left( {P^{*} \frac{{\partial \:P_{w} }}{{\partial \:x}} + P_{w} \frac{{\partial \:P^{*} }}{{\partial \:x}}} \right) + vP_{w} \frac{{\partial \:P^{*} }}{{\partial \:y}} \hfill \\ = D_{p} \left( {P^{*} \frac{{\partial \:^{2} P_{w} }}{{\partial \:x^{2} }} + 2\frac{{\partial \:P_{w} }}{{\partial \:x}}\frac{{\partial \:P^{*} }}{{\partial \:x}} + P_{w} \frac{{\partial \:^{2} P^{*} }}{{\partial \:x^{2} }} + P_{w} \frac{{\partial \:^{2} P^{*} }}{{\partial \:y^{2} }}} \right) \hfill \\ - \alpha \:P^{*} P_{w} + \gamma \:\Delta \:T\theta \: + \zeta \:^{*} \Delta \:CC^{*} + \delta \:(H_{1}^{{*2}} H_{{1w}}^{2} + H_{2}^{{*2}} H_{{2w}}^{2} ) \hfill \\ \end{gathered}$$

The normalized boundary conditions are described hereafter:

$$\:y=0$$

$$\:{u}^{*}=1,\:v={v}_{0},\:{H}_{1}^{*}=1,\:{H}_{2}^{*}=1,{\:R}^{*}=1,{P}^{*}=1,\theta\:=1,\:\:{C}^{*}=1,{\:\:N}^{*}=1.$$

$$\:y\to\:\infty\:$$

22 $$\:{u}^{*}=0,\:{H}_{1}^{*}=0,\:{H}_{2}^{*}=0,{\:R}^{*}=0,{P}^{*}=0,\theta\:=0,\:\:{C}^{*}=0,{\:\:N}^{*}=0.$$

## Invariance of the mathematical model

A two-parameter group of transformations is commonly applied to a set of partial differential equations (PDEs) to modify the system’s invariant group. Similarity solutions can be derived using the parameters ($\:{\alpha\:}_{1},{\alpha\:}_{2}$), which represent the continuous symmetries of the system. Creating the similarity variables, $\:\eta\:$, helps in simplifying the mathematical model into a system of ordinary differential equations (ODEs). This approach does not only facilitate the procedures of solving complicated nonlinear PDEs, but also enhances the understanding of the system’s fundamental symmetries and parameters.

### Similarity analysis of the mathematical model

Let’s assume that the group structure is as follows:

23 $$\:G:\stackrel{-}{S}={K}^{s}\left({\alpha\:}_{1},{\alpha\:}_{2}\right)S+{Q}^{s}({\alpha\:}_{1},{\alpha\:}_{2})$$

Similarity transformations reduce the PDE system into a more tractable ODE system based on scaling symmetries. The symbol $\:S$ stands for the system variables, while the real values of $\:{K}^{s}$ and $\:{Q}^{s}$ corresponds to the differential coefficient functions. Partial derivatives are typically expressed as follows:

24 $$\:\left.\begin{array}{c}{\stackrel{-}{S}}_{\stackrel{-}{i}}=\left(\frac{{K}^{s}}{{K}^{i}}\right){S}_{i\:\:\:\:\:}\\\:{\stackrel{-}{S}}_{\stackrel{-}{ij}}=\left(\frac{{K}^{s}}{{K}^{i}{K}^{j}}\right){S}_{ij}\end{array}\right\}i=x,y,t\:and\:j=x,y,t$$

#### Analysis of the problem

Using the previously mentioned relations (23) and (24), Eq. (13), becomes:

25 $$\:\stackrel{-}{{u}^{*}}\frac{\partial\:\stackrel{-}{{u}_{w}}}{\partial\:\stackrel{-}{x}}+\stackrel{-}{{u}_{w}}\frac{\partial\:\stackrel{-}{{u}^{*}}}{\partial\:\stackrel{-}{x}}+\frac{\partial\:\stackrel{-}{v}}{\partial\:\stackrel{-}{y}}={H}_{1}\left({\alpha\:}_{1},{\alpha\:}_{2}\right)\left[{u}^{*}\frac{\partial\:{u}_{w}}{\partial\:x}+{u}_{w}\frac{\partial\:{u}^{*}}{\partial\:x}+\frac{\partial\:v}{\partial\:y}\right]$$

The Invariance parameter is $\:{H}_{1}\left({\alpha\:}_{1},{\alpha\:}_{2}\right)$, while and the transformed variables are indicated by the slashes. Invoking Eqs. (23) and (24) and substituting into (25) results in:

26 $$\:\frac{{k}^{{u}^{*}}{k}^{{u}_{w}}}{{k}^{x}}{u}^{*}\frac{\partial\:{u}_{w}}{\partial\:x}+\frac{{k}^{{u}^{*}}{k}^{{u}_{w}}}{{k}^{x}}{u}_{w}\frac{\partial\:{u}^{*}}{\partial\:x}+\frac{{k}^{v}}{{k}^{y}}\frac{\partial\:v}{\partial\:y}={H}_{1}\left({\alpha\:}_{1},{\alpha\:}_{2}\right)\left[{u}^{*}\frac{\partial\:{u}_{w}}{\partial\:x}+{u}_{w}\frac{\partial\:{u}^{*}}{\partial\:x}+\frac{\partial\:v}{\partial\:y}\right]$$

Equations (14–21), similarly, are transformed to:

27 $$\begin{gathered} \:\frac{{k^{{u^{*} }} k^{{u_{w} }} }}{{k^{t} }}u^{*} \frac{{\partial \:u_{w} }}{{\partial \:t}} + \frac{{k^{{u^{*} }} k^{{u_{w} }} }}{{k^{t} }}u_{w} \frac{{\partial \:u^{*} }}{{\partial \:t}} \hfill \\ + k^{{u^{*} }} k^{{u_{w} }} u^{*} u_{w} \left( {\frac{{k^{{u^{*} }} k^{{u_{w} }} }}{{k^{x} }}u^{*} \frac{{\partial \:u_{w} }}{{\partial \:x}} + \frac{{k^{{u^{*} }} k^{{u_{w} }} }}{{k^{x} }}u_{w} \frac{{\partial \:u^{*} }}{{\partial \:x}}} \right) \hfill \\ + k^{v} v\left( {\frac{{k^{{u^{*} }} k^{{u_{w} }} }}{{k^{y} }}u_{w} \frac{{\partial \:u^{*} }}{{\partial \:y}}} \right) - a_{1} \left( {\frac{{k^{{u^{*} }} k^{{u_{w} }} }}{{\left( {k^{y} } \right)^{2} }}u_{w} \frac{{\partial \:^{2} u^{*} }}{{\partial \:y^{2} }}} \right) + k^{{u^{*} }} k^{{u_{w} }} a_{2} u^{*} u_{w} \hfill \\ - k^{{\theta \:}} a_{3} \Delta \:T\theta \: + k^{{c^{*} }} a_{4} \Delta \:CC^{*} + k^{{N^{*} }} a_{5} \Delta \:NN^{*} \hfill \\ - k^{{H_{2}^{*} }} k^{{H_{{2w}} }} a_{6} H_{2}^{*} H_{{2w}} \left( {\frac{{k^{{H_{1}^{*} }} k^{{H_{{1w}} }} }}{{k^{x} }}H_{1}^{*} \frac{{\partial \:H_{{1w}} }}{{\partial \:x}} + \frac{{k^{{H_{1}^{*} }} k^{{H_{{1w}} }} }}{{k^{x} }}H_{{1w}} \frac{{\partial \:H_{1}^{*} }}{{\partial \:x}} + \frac{{k^{{H_{1}^{*} }} k^{{H_{{1w}} }} }}{{k^{y} }}H_{{1w}} \frac{{\partial \:H_{1}^{*} }}{{\partial \:y}}} \right) \hfill \\ = H_{1} \left( {\alpha \:_{1} ,\alpha \:_{2} } \right)\left[ \begin{gathered} u^{*} \frac{{\partial \:u_{w} }}{{\partial \:t}} + u_{w} \frac{{\partial \:u^{*} }}{{\partial \:t}} + u^{*} u_{w} \left( {u^{*} \frac{{\partial \:u_{w} }}{{\partial \:x}} + u_{w} \frac{{\partial \:u^{*} }}{{\partial \:x}}} \right) + v\left( {u_{w} \frac{{\partial \:u^{*} }}{{\partial \:y}}} \right) \hfill \\ - a_{1} \left( {u_{w} \frac{{\partial \:^{2} u^{*} }}{{\partial \:y^{2} }}} \right) + a_{2} u^{*} u_{w} - a_{3} \Delta \:T\theta \: + a_{4} \Delta \:CC^{*} + a_{5} \Delta \:NN^{*} \hfill \\ - a_{6} H_{2}^{*} H_{{2w}} \left( {H_{1}^{*} \frac{{\partial \:H_{{1w}} }}{{\partial \:x}} + H_{{1w}} \frac{{\partial \:H_{1}^{*} }}{{\partial \:x}} + H_{{1w}} \frac{{\partial \:H_{1}^{*} }}{{\partial \:y}}} \right) \hfill \\ \end{gathered} \right] \hfill \\ \end{gathered}$$

28 $$\begin{gathered} \frac{{k^{\theta } }}{{k^{t} }}\frac{{\partial \theta }}{{\partial t}} + \frac{{k^{{u^{*} }} k^{{u_{w} }} k^{\theta } }}{{k^{x} }}u^{*} u_{w} \frac{{\partial \theta }}{{\partial x}} + \frac{{k^{v} k^{\theta } }}{{k^{y} }}v\frac{{\partial \theta }}{{\partial y}} - a_{7} \frac{{k^{\theta } }}{{\left( {k^{y} } \right)^{2} }}\frac{{\partial ^{2} \theta }}{{\partial y^{2} }} \hfill \\ - \left[ {a_{8} \Delta C\frac{{k^{{C^{*} }} k^{\theta } }}{{\left( {k^{y} } \right)^{2} }}\frac{{\partial C^{*} }}{{\partial y}}\frac{{\partial \theta }}{{\partial y}} + a_{9} \Delta T\left( {\frac{{k^{\theta } }}{{k^{y} }}\frac{{\partial \theta }}{{\partial y}}} \right)^{2} } \right] - a_{{10}} k^{\theta } \theta \hfill \\ = H_{1} \left( {\alpha _{1} ,\alpha _{2} } \right)\left[ \begin{gathered} \frac{{\partial \theta }}{{\partial t}} + u^{*} u_{w} \frac{{\partial \theta }}{{\partial x}} + v\frac{{\partial \theta }}{{\partial y}} - a_{7} \frac{{\partial ^{2} \theta }}{{\partial y^{2} }} \hfill \\ - \left[ {a_{8} \Delta C\frac{{\partial C^{*} }}{{\partial y}}\frac{{\partial \theta }}{{\partial y}} + a_{9} \Delta T\left( {\frac{{\partial \theta }}{{\partial y}}} \right)^{2} } \right] - a_{{10}} \theta \hfill \\ \end{gathered} \right]~ \hfill \\ \end{gathered}$$

29 $$\begin{gathered} \frac{{k^{{C^{*} }} }}{{k^{t} }}\frac{{\partial C^{*} }}{{\partial t}} + \frac{{k^{{u^{*} }} k^{{u_{w} }} k^{{C^{*} }} }}{{k^{x} }}u^{*} u_{w} \frac{{\partial C^{*} }}{{\partial x}} + \frac{{k^{v} k^{{C^{*} }} }}{{k^{y} }}v\frac{{\partial C^{*} }}{{\partial y}} - D_{B} \left( {\frac{{k^{{C^{*} }} }}{{\left( {k^{x} } \right)^{2} }}\frac{{\partial ^{2} C^{*} }}{{\partial x^{2} }} + \frac{{k^{{C^{*} }} }}{{\left( {k^{y} } \right)^{2} }}\frac{{\partial ^{2} C^{*} }}{{\partial y^{2} }}} \right) \hfill \\ = H_{1} \left( {\alpha _{1} ,\alpha _{2} } \right)\left[ {\frac{{\partial C^{*} }}{{\partial t}} + u^{*} u_{w} \frac{{\partial C^{*} }}{{\partial x}} + v\frac{{\partial C^{*} }}{{\partial y}} - D_{B} \left( {\frac{{\partial ^{2} C^{*} }}{{\partial x^{2} }} + \frac{{\partial ^{2} C^{*} }}{{\partial y^{2} }}} \right)} \right] \hfill \\ \end{gathered}$$

30 $$\begin{gathered} \frac{{k^{{N^{*} }} }}{{k^{t} }}\frac{{\partial N^{*} }}{{\partial t}} + \frac{{k^{{u^{*} }} k^{{u_{w} }} k^{{N^{*} }} }}{{k^{x} }}u^{*} u_{w} \frac{{\partial N^{*} }}{{\partial x}} + \frac{{k^{v} k^{{N^{*} }} }}{{k^{y} }}v\frac{{\partial N^{*} }}{{\partial y}} + a_{{11}} \left[ {\frac{{k^{{N^{*} }} }}{{k^{y} }}\frac{{\partial N^{*} }}{{\partial y}}} \right] - a_{{12}} \left( {\frac{{k^{{N^{*} }} }}{{\left( {k^{x} } \right)^{2} }}\frac{{\partial ^{2} N^{*} }}{{\partial x^{2} }} + \frac{{k^{{N^{*} }} }}{{\left( {k^{y} } \right)^{2} }}\frac{{\partial ^{2} N^{*} }}{{\partial y^{2} }}} \right) \hfill \\ = H_{1} \left( {\alpha _{1} ,\alpha _{2} } \right)\left[ {\frac{{\partial N^{*} }}{{\partial t}} + u^{*} u_{w} \frac{{\partial N^{*} }}{{\partial x}} + v\frac{{\partial N^{*} }}{{\partial y}} + a_{{11}} \left[ {\frac{{\partial N^{*} }}{{\partial y}}} \right] - a_{{12}} \left( {\frac{{\partial ^{2} N^{*} }}{{\partial x^{2} }} + \frac{{\partial ^{2} N^{*} }}{{\partial y^{2} }}} \right)} \right]~~ \hfill \\ \end{gathered}$$

31 $$\begin{gathered} \:\frac{{k^{{H_{1}^{*} }} k^{{H_{{1w}} }} }}{{k^{t} }}H_{1}^{*} \frac{{\partial \:H_{{1w}} }}{{\partial \:t}} + \frac{{k^{{H_{1}^{*} }} k^{{H_{{1w}} }} }}{{k^{t} }}H_{{1w}} \frac{{\partial \:H_{1}^{*} }}{{\partial \:t}} \hfill \\ + k^{{u^{*} }} k^{{u_{w} }} u^{*} u_{w} \left( {\frac{{k^{{H_{1}^{*} }} k^{{H_{{1w}} }} }}{{k^{x} }}H_{1}^{*} \frac{{\partial \:H_{{1w}} }}{{\partial \:x}} + \frac{{k^{{H_{1}^{*} }} k^{{H_{{1w}} }} }}{{k^{x} }}H_{{1w}} \frac{{\partial \:H_{1}^{*} }}{{\partial \:x}}} \right) \hfill \\ + \frac{{k^{v} k^{{H_{1}^{*} }} k^{{H_{{1w}} }} }}{{k^{y} }}vH_{{1w}} \frac{{\partial \:H_{1}^{*} }}{{\partial \:y}} - \eta \:_{0} \left( {\frac{{k^{{H_{1}^{*} }} k^{{H_{{1w}} }} }}{{\left( {k^{y} } \right)^{2} }}H_{{1w}} \frac{{\partial \:^{2} H_{1}^{*} }}{{\partial \:y^{2} }}} \right) \hfill \\ = H_{1} \left( {\alpha \:_{1} ,\alpha \:_{2} } \right)\left[ \begin{gathered} H_{1}^{*} \frac{{\partial \:H_{{1w}} }}{{\partial \:t}} + H_{{1w}} \frac{{\partial \:H_{1}^{*} }}{{\partial \:t}} + u^{*} u_{w} \left( {H_{1}^{*} \frac{{\partial \:H_{{1w}} }}{{\partial \:x}} + H_{{1w}} \frac{{\partial \:H_{1}^{*} }}{{\partial \:x}}} \right) \hfill \\ + vH_{{1w}} \frac{{\partial \:H_{1}^{*} }}{{\partial \:y}} - \eta \:_{0} \left( {H_{{1w}} \frac{{\partial \:^{2} H_{1}^{*} }}{{\partial \:y^{2} }}} \right) \hfill \\ \end{gathered} \right] \hfill \\ \end{gathered}$$

32 $$\begin{gathered} \:\frac{{k^{{H_{2}^{*} }} k^{{H_{{2w}} }} }}{{k^{t} }}H_{2}^{*} \frac{{\partial \:H_{{2w}} }}{{\partial \:t}} + \frac{{k^{{H_{2}^{*} }} k^{{H_{{2w}} }} }}{{k^{t} }}H_{{2w}} \frac{{\partial \:H_{2}^{*} }}{{\partial \:t}} \hfill \\ + k^{{u^{*} }} k^{{u_{w} }} u^{*} u_{w} \left( {\frac{{k^{{H_{2}^{*} }} k^{{H_{{2w}} }} }}{{k^{x} }}H_{2}^{*} \frac{{\partial \:H_{{2w}} }}{{\partial \:x}} + \frac{{k^{{H_{2}^{*} }} k^{{H_{{2w}} }} }}{{k^{x} }}H_{{2w}} \frac{{\partial \:H_{2}^{*} }}{{\partial \:x}}} \right) \hfill \\ + \frac{{k^{v} k^{{H_{2}^{*} }} k^{{H_{{2w}} }} }}{{k^{y} }}vH_{{2w}} \frac{{\partial \:H_{2}^{*} }}{{\partial \:y}} - \eta \:_{0} \left( {\frac{{k^{{H_{2}^{*} }} k^{{H_{{2w}} }} }}{{\left( {k^{y} } \right)^{2} }}H_{{2w}} \frac{{\partial \:^{2} H_{2}^{*} }}{{\partial \:y^{2} }}} \right) \hfill \\ = H_{1} \left( {\alpha \:_{1} ,\alpha \:_{2} } \right)\left[ \begin{gathered} H_{2}^{*} \frac{{\partial \:H_{{2w}} }}{{\partial \:t}} + H_{{2w}} \frac{{\partial \:H_{2}^{*} }}{{\partial \:t}} + u^{*} u_{w} \left( {H_{2}^{*} \frac{{\partial \:H_{{2w}} }}{{\partial \:x}} + H_{{2w}} \frac{{\partial \:H_{2}^{*} }}{{\partial \:x}}} \right) \hfill \\ + vH_{{2w}} \frac{{\partial \:H_{2}^{*} }}{{\partial \:y}} - \eta \:_{0} \left( {H_{{2w}} \frac{{\partial \:^{2} H_{2}^{*} }}{{\partial \:y^{2} }}} \right) \hfill \\ \end{gathered} \right] \hfill \\ \end{gathered}$$

33 $$\begin{gathered} \:\frac{{k^{{R^{*} }} k^{{R_{w} }} }}{{k^{t} }}R^{*} \frac{{\partial \:R_{w} }}{{\partial \:t}} + \frac{{k^{{R^{*} }} k^{{R_{w} }} }}{{k^{t} }}R_{w} \frac{{\partial \:R^{*} }}{{\partial \:t}} \hfill \\ + k^{{u^{*} }} k^{{u_{w} }} u^{*} u_{w} \left( {\frac{{k^{{R^{*} }} k^{{R_{w} }} }}{{k^{x} }}R^{*} \frac{{\partial \:R_{w} }}{{\partial \:x}} + \frac{{k^{{R^{*} }} k^{{R_{w} }} }}{{k^{x} }}R_{w} \frac{{\partial \:R^{*} }}{{\partial \:x}}} \right) \hfill \\ + \frac{{k^{v} k^{{R^{*} }} k^{{R_{w} }} }}{{k^{y} }}vR_{w} \frac{{\partial \:R^{*} }}{{\partial \:y}} - D_{r} \left( \begin{gathered} \frac{{k^{{R^{*} }} k^{{R_{w} }} }}{{\left( {k^{x} } \right)^{2} }}R^{*} \frac{{\partial \:^{2} R_{w} }}{{\partial \:x^{2} }} + \frac{{k^{{R^{*} }} k^{{R_{w} }} }}{{\left( {k^{x} } \right)^{2} }}\frac{{\partial \:R_{w} }}{{\partial \:x}}\frac{{\partial \:R^{*} }}{{\partial \:x}} \hfill \\ + \frac{{k^{{R^{*} }} k^{{R_{w} }} }}{{\left( {k^{x} } \right)^{2} }}R_{w} \frac{{\partial \:^{2} R^{*} }}{{\partial \:x^{2} }} + \frac{{k^{{R^{*} }} k^{{R_{w} }} }}{{\left( {k^{x} } \right)^{2} }}\frac{{\partial \:R^{*} }}{{\partial \:x}}\frac{{\partial \:R_{w} }}{{\partial \:x}} \hfill \\ + \frac{{k^{{R^{*} }} k^{{R_{w} }} }}{{\left( {k^{y} } \right)^{2} }}R_{w} \frac{{\partial \:^{2} R^{*} }}{{\partial \:y^{2} }} \hfill \\ \end{gathered} \right) \hfill \\ + k_{r} \left( {c_{1}^{*} \:k^{{H_{1}^{{*{\kern 1pt} }} }} k^{{H_{{1w}} }} \:H_{1}^{{*\:}} H_{{1w}} - c_{2} ^{*} k^{{H_{2}^{*} }} k^{{H_{{2w}} }} H_{2}^{*} H_{{2w}} } \right) \hfill \\ = H_{1} \left( {\alpha \:_{1} ,\alpha \:_{2} } \right)\left[ \begin{gathered} R^{*} \frac{{\partial \:R_{w} }}{{\partial \:t}} + R_{w} \frac{{\partial \:R^{*} }}{{\partial \:t}} + u^{*} u_{w} \left( {R^{*} \frac{{\partial \:R_{w} }}{{\partial \:x}} + R_{w} \frac{{\partial \:R^{*} }}{{\partial \:x}}} \right) \hfill \\ + vR_{w} \frac{{\partial \:R^{*} }}{{\partial \:y}} - D_{r} \left( \begin{gathered} R^{*} \frac{{\partial \:^{2} R_{w} }}{{\partial \:x^{2} }} + \frac{{\partial \:R_{w} }}{{\partial \:x}}\frac{{\partial \:R^{*} }}{{\partial \:x}} \hfill \\ + R_{w} \frac{{\partial \:^{2} R^{*} }}{{\partial \:x^{2} }} + \frac{{\partial \:R^{*} }}{{\partial \:x}}\frac{{\partial \:R_{w} }}{{\partial \:x}} + R_{w} \frac{{\partial \:^{2} R^{*} }}{{\partial \:y^{2} }} \hfill \\ \end{gathered} \right) \hfill \\ + k_{r} \left( {c_{1}^{*} H_{1}^{*} H_{{1w}} - c_{2}^{*} H_{2}^{*} H_{{2w}} } \right) \hfill \\ \end{gathered} \right] \hfill \\ \end{gathered}$$

34 $$\begin{gathered} \:\frac{{k^{{P^{*} }} k^{{P_{w} }} }}{{k^{t} }}P^{*} \frac{{\partial \:P_{w} }}{{\partial \:t}} + \frac{{k^{{P^{*} }} k^{{P_{w} }} }}{{k^{t} }}P_{w} \frac{{\partial \:P^{*} }}{{\partial \:t}} \hfill \\ + k^{{u^{*} }} k^{{u_{w} }} u^{*} u_{w} \left( {\frac{{k^{{P^{*} }} k^{{P_{w} }} }}{{k^{x} }}P^{*} \frac{{\partial \:P_{w} }}{{\partial \:x}} + \frac{{k^{{P^{*} }} k^{{P_{w} }} }}{{k^{x} }}P_{w} \frac{{\partial \:P^{*} }}{{\partial \:x}}} \right) \hfill \\ + \frac{{k^{v} k^{{P^{*} }} k^{{P_{w} }} }}{{k^{y} }}vP_{w} \frac{{\partial \:P^{*} }}{{\partial \:y}} - D_{p} \left( \begin{gathered} \frac{{k^{{P^{*} }} k^{{P_{w} }} }}{{\left( {k^{x} } \right)^{2} }}P^{*} \frac{{\partial \:^{2} P_{w} }}{{\partial \:x^{2} }} + \frac{{k^{{P^{*} }} k^{{P_{w} }} }}{{\left( {k^{x} } \right)^{2} }}\frac{{\partial \:P_{w} }}{{\partial \:x}}\frac{{\partial \:P^{*} }}{{\partial \:x}} \hfill \\ + \frac{{k^{{P^{*} }} k^{{P_{w} }} }}{{\left( {k^{x} } \right)^{2} }}P_{w} \frac{{\partial \:^{2} P^{*} }}{{\partial \:x^{2} }} + \frac{{k^{{P^{*} }} k^{{P_{w} }} }}{{\left( {k^{x} } \right)^{2} }}\frac{{\partial \:P^{*} }}{{\partial \:x}}\frac{{\partial \:P_{w} }}{{\partial \:x}} \hfill \\ + \frac{{k^{{P^{*} }} k^{{P_{w} }} }}{{\left( {k^{y} } \right)^{2} }}P_{w} \frac{{\partial \:^{2} P^{*} }}{{\partial \:y^{2} }} \hfill \\ \end{gathered} \right) \hfill \\ + k^{{P^{*} }} k^{{P_{w} }} \alpha \:P^{*} P_{w} - k^{{\theta \:}} \gamma \:\Delta \:T\theta \: - k^{{C^{*} }} \zeta \:^{*} \Delta \:CC^{*} \hfill \\ - \delta \:\left( {\left( {k^{{H_{1}^{*} }} } \right)^{2} \left( {k^{{H_{{1w}} }} } \right)^{2} H_{1}^{{*2}} H_{{1w}}^{2} + \left( {k^{{H_{2}^{*} }} } \right)^{2} \left( {k^{{H_{{2w}} }} } \right)^{2} H_{2}^{{*2}} H_{{2w}}^{2} } \right) \hfill \\ = H_{1} \left( {\alpha \:_{1} ,\alpha \:_{2} } \right)\left[ \begin{gathered} P^{*} \frac{{\partial \:P_{w} }}{{\partial \:t}} + P_{w} \frac{{\partial \:P^{*} }}{{\partial \:t}} + u^{*} u_{w} \left( {P^{*} \frac{{\partial \:P_{w} }}{{\partial \:x}} + P_{w} \frac{{\partial \:P^{*} }}{{\partial \:x}}} \right) \hfill \\ + vP_{w} \frac{{\partial \:P^{*} }}{{\partial \:y}} - D_{p} \left( \begin{gathered} P^{*} \frac{{\partial \:^{2} P_{w} }}{{\partial \:x^{2} }} + \frac{{\partial \:P_{w} }}{{\partial \:x}}\frac{{\partial \:P^{*} }}{{\partial \:x}} + P_{w} \frac{{\partial \:^{2} P^{*} }}{{\partial \:x^{2} }} \hfill \\ + \frac{{\partial \:P^{*} }}{{\partial \:x}}\frac{{\partial \:P_{w} }}{{\partial \:x}} + P_{w} \frac{{\partial \:^{2} P^{*} }}{{\partial \:y^{2} }} \hfill \\ \end{gathered} \right) \hfill \\ + \alpha \:P^{*} P_{w} - \gamma \:\Delta \:T\theta \: - \zeta \:^{*} \Delta \:CC^{*} - \delta \:\left( {H_{1}^{{*2}} H_{{1w}}^{2} + H_{2}^{{*2}} H_{{2w}}^{2} } \right) \hfill \\ \end{gathered} \right] \hfill \\ \end{gathered}$$

The invariance criterion leads to the following results:

$$\:{K}^{{u}_{w}}{K}^{{u}^{*}}={K}^{x}={K}^{v}={K}^{{R}^{*}}{K}^{{R}_{w}}={K}^{{P}^{*}}{K}^{{P}_{w}}={K}^{y}={K}^{t}={K}^{{N}^{*}}={K}^{{C}^{*}}={K}^{\theta\:}={K}^{{{H}_{2}}_{w}}{K}^{{H}_{2}^{*}}={K}^{{{H}_{1}}_{w}}{K}^{{H}_{1}^{*}}=1$$

35 $$\:{Q}^{{u}_{w}}={Q}^{{u}^{*}}={Q}^{{P}_{w}}={Q}^{{P}^{*}}={Q}^{{R}_{w}}={Q}^{{R}^{*}}={Q}^{{C}^{*}}={Q}^{\theta\:}={Q}^{{H}_{1}^{*}}={Q}^{{H}_{2}^{*}}={Q}^{{{H}_{1}}_{w}}={Q}^{{{H}_{2}}_{w}}={Q}^{{N}^{*}}={Q}^{v}={Q}^{t}=0$$

Ultimately, the group’s structure, G, has the following form:

36 $$\:G:\left\{\begin{array}{c}{G}_{1}\left\{\begin{array}{c}\stackrel{-}{x}=x+{Q}^{x}\\\:\stackrel{-}{y}=y+{Q}^{y}\\\:\stackrel{-}{t}=t+{Q}^{t}\end{array}\right.\\\:{G}_{2}\left\{\begin{array}{c}\stackrel{-}{{C}^{*}}={C}^{*}\\\:\stackrel{-}{\theta\:}=\theta\:\\\:\stackrel{-}{{N}^{*}}={N}^{*}\\\:{\stackrel{-}{u}}_{w}={K}^{{u}_{w}}{u}_{w}\\\:{\stackrel{-}{p}}_{w}={K}^{{p}_{w}}{p}_{w}\\\:{\stackrel{-}{R}}_{w}={K}^{{R}_{w}}{R}_{w}\\\:\stackrel{-}{{{H}_{1}}_{w}}={K}^{{{H}_{1}}_{w}}{{H}_{1}}_{w}\\\:\stackrel{-}{{{H}_{2}}_{w}}={K}^{{{H}_{2}}_{w}}{{H}_{2}}_{w}\\\:\stackrel{-}{{u}^{*}}={k}^{{u}^{*}}{u}^{*}\\\:\stackrel{-}{{P}^{*}}={k}^{{P}^{*}}{P}^{*}\\\:\stackrel{-}{{R}^{*}}={k}^{{R}^{*}}{R}^{*}\\\:\stackrel{-}{v}=v\\\:\stackrel{-}{{H}_{1}^{*}}={K}^{{H}_{1}^{*}}{H}_{1}^{*}\\\:\stackrel{-}{{H}_{2}^{*}}={K}^{{H}_{2}^{*}}{H}_{2}^{*}\end{array}\right.\end{array}\right.$$

### The transformation of the whole system’s variables

The following formula is used to transform both independent and dependent variables^48,50,51^.

37 $$\:{\sum\:}_{i=1}^{17}\left({\gamma\:}_{i}{S}_{i}+{\delta\:}_{i}\right)\frac{\partial\:{q}_{i}}{\partial\:{S}_{i}}=0$$

This formula depicts a connection that frequently appears in systems analysis, especially when reducing complexity by changing dependent variables.

#### Methodology overview

- Single similarity variable: The objective is to use a single similarity variable to reduce the number of independent variables. This aids simplifying the analysis and lowering the problem’s dimensionality.
- Initial and new variables:
- Initial dependent variables: $\:{S}_{i}$, (for $\:i=1,\dots\:,17$)
- New invariant dependent variables:

$$\:{u}^{*},{u}_{w},v,{c}^{*}\:,\theta\:,{N}^{*},{H}_{1}^{*},{{H}_{1}}_{w},{H}_{2}^{*},{{H}_{2}}_{w},{P}^{*},{P}_{w},{R}^{*},{R}_{w}$$

#### Coefficients definition

The following is a definition of the coefficients $\:{\gamma\:}_{i}\:and\:{\delta\:}_{i}$:

38 $$\:\left\{\begin{array}{c}{\gamma\:}_{i}=\frac{\partial\:{K}^{{S}_{i}}\left(\alpha\:\right)}{\partial\:\alpha\:}\:\\\:{\delta\:}_{i}=\frac{\partial\:{Q}^{{S}_{i}}\left(\alpha\:\right)}{\partial\:\alpha\:}\end{array}\right.$$

### The independent variables’ transformation

The independent variables $\:(x,\:y,\:and\:t)$ have been combined into a single similarity variable using Eq. (37). The variables are supplied by: Using the same methods as outlined in references^52–56^.

- 39 $$\:\eta\:=\pi\:\left(x,t\right)y$$

Additionally, the dependent variables have been modified to:

- $$\:{u}^{*}=\omega\:\left(x,t\right)F\left(\eta\:\right)$$
- $$\:v=v\left(\eta\:\right)$$
- $$\:{R}^{*}=\zeta\:\left(x,t\right)E\left(\eta\:\right)$$
- $$\:{P}^{*}=\epsilon\:\left(x,t\right)S\left(\eta\:\right)$$
- $$\:{H}_{1}^{*}={\Gamma\:}\left(x,t\right)H\left(\eta\:\right)$$
- $$\:{H}_{2}^{*}={\uplambda\:}\left(x,t\right)J\left(\eta\:\right)$$
- $$\:{u}_{w}={u}_{w}(x,t)$$
- $$\:{R}_{w}={R}_{w}\left(x,t\right)$$
- $$\:{P}_{w}={P}_{w}\left(x,t\right)$$
- $$\:{{H}_{1}}_{w}={{H}_{1}}_{w}\left(x,t\right)$$
- $$\:{{H}_{2}}_{w}={{H}_{2}}_{w}\left(x,t\right)$$
- $$\:\theta\:=\theta\:\left(\eta\:\right)$$
- $$\:{C}^{*}={C}^{*}\left(\eta\:\right)$$
- 40 $$\:{N}^{*}={\uppsi\:}\left(x,t\right)g\left(\eta\:\right)$$

Where$$\:\pi\:\left(x,t\right),\omega\:\left(x,t\right),\zeta\:\left(x,t\right),\epsilon\:\left(x,t\right),{\Gamma\:}\left(x,t\right),{\uplambda\:}\left(x,t\right),{u}_{w}\left(x,t\right),{R}_{w}\left(x,t\right),{P}_{w}\left(x,t\right),{{H}_{1}}_{w}\left(x,t\right),$$

$\:{{H}_{2}}_{w}\left(x,t\right)\:and\:{\uppsi\:}\left(x,t\right)$ represent arbitrary functions that will be assessed during the reduction procedures.

The following is the tranformed system of the original model, (13)–(21),:

41 $$\:\frac{\omega\:\frac{\partial\:{u}_{w}}{\partial\:x}}{\pi\:}F+\frac{{u}_{w}\omega\:\frac{\partial\:\pi\:}{\partial\:x}}{{\pi\:}^{2}}F{\prime\:}\eta\:+\frac{{u}_{w}\frac{\partial\:\omega\:}{\partial\:x}}{\pi\:}F+v{\prime\:}=0$$

42 $$\begin{gathered} \:\frac{{\frac{{\partial \:u_{w} }}{{\partial \:t}}}}{{a_{1} u_{w} \pi \:^{2} }}F + \frac{{\frac{{\partial \:\pi \:}}{{\partial \:t}}}}{{a_{1} \pi \:^{3} }}\eta \:F\prime \: + \frac{{\frac{{\partial \:\omega \:}}{{\partial \:t}}}}{{a_{1} \omega \:\pi \:^{2} }}F + \frac{{\omega \:\frac{{\partial \:u_{w} }}{{\partial \:x}}}}{{a_{1} \pi \:^{2} }}F^{2} \hfill \\ + \frac{{\omega \:u_{w} \frac{{\partial \:\pi \:}}{{\partial \:x}}}}{{a_{1} \pi \:^{3} }}\eta \:F\prime \:F + \frac{{u_{w} \frac{{\partial \:\omega \:}}{{\partial \:x}}}}{{a_{1} \pi \:^{2} }}F^{2} + \frac{1}{{a_{1} \pi \:}}vF\prime \: \hfill \\ = F\prime \:\prime \: - \frac{{a_{2} }}{{a_{1} \pi \:^{2} }}F + \frac{{a_{3} \Delta \:T}}{{a_{1} u_{w} \omega \:\pi \:^{2} }}\theta \: - \frac{{a_{4} \Delta \:C}}{{a_{1} u_{w} \omega \:\pi \:^{2} }}C^{*} \hfill \\ - \frac{{a_{5} \Delta \:N\psi \:}}{{a_{1} u_{w} \omega \:\pi \:^{2} }}g + \frac{{a_{6} \lambda \:H_{{2w}} \Gamma \:\frac{{\partial \:H_{{1w}} }}{{\partial \:x}}}}{{a_{1} u_{w} \omega \:\pi \:^{2} }}JH + \frac{{a_{6} \lambda \:H_{{2w}} H_{{1w}} \Gamma \:\frac{{\partial \:\pi \:}}{{\partial \:x}}}}{{a_{1} u_{w} \omega \:\pi \:^{3} }}JH\prime \:\eta \: \hfill \\ + \frac{{a_{6} \lambda \:H_{{2w}} H_{{1w}} \frac{{\partial \:\Gamma \:}}{{\partial \:x}}}}{{a_{1} u_{w} \omega \:\pi \:^{2} }}JH + \frac{{a_{6} \lambda \:H_{{2w}} H_{{1w}} \Gamma \:}}{{a_{1} u_{w} \omega \:\pi \:}}JH\prime \: \hfill \\ \end{gathered}$$

43 $$\:\frac{\frac{\partial\:\pi\:}{\partial\:t}}{{a}_{7}{\pi\:}^{3}}\theta\:{\prime\:}\eta\:+\frac{\omega\:{u}_{w}\frac{\partial\:\pi\:}{\partial\:x}}{{a}_{7}{\pi\:}^{3}}F\theta\:{\prime\:}\eta\:+\frac{1}{{a}_{7}\pi\:}v\theta\:{\prime\:}=\theta\:{\prime\:}{\prime\:}+\frac{{a}_{8}{\Delta\:}C}{{a}_{7}}{c}^{{*}^{{\prime\:}}}\theta\:{\prime\:}+\frac{{a}_{9}{\Delta\:}T}{{a}_{7}}{\theta\:{\prime\:}}^{2}+\frac{{a}_{10}}{{a}_{7}{\pi\:}^{2}}\theta\:$$

44 $$\:\frac{\frac{\partial\:\pi\:}{\partial\:t}}{{D}_{B}{\pi\:}^{3}}{C}^{*}{\prime\:}\eta\:+\frac{\omega\:{u}_{w}\frac{\partial\:\pi\:}{\partial\:x}}{{D}_{B}{\pi\:}^{3}}{FC}^{*}{\prime\:}\eta\:+\frac{1}{{D}_{B}\pi\:}v{C}^{*}{\prime\:}=\frac{\frac{{\partial\:}^{2}\pi\:}{\partial\:{x}^{2}}}{{\pi\:}^{3}}{C}^{*}{\prime\:}\eta\:+\frac{{\left(\frac{\partial\:\pi\:}{\partial\:x}\right)}^{2}}{{\pi\:}^{4}}{C}^{*}{\prime\:}{\eta\:}^{2}+{C}^{*}{\prime\:}{\prime\:}$$

45 $$\begin{gathered} \frac{{\frac{{\partial \pi }}{{\partial t}}}}{{a_{{12}} \pi ^{3} }}g'\eta + \frac{{\frac{{\partial \psi }}{{\partial t}}}}{{a_{{12}} \psi \pi ^{2} }}g + \frac{{\omega u_{w} \frac{{\partial \pi }}{{\partial x}}}}{{a_{{12}} \pi ^{3} }}g'\eta F + \frac{{\omega u_{w} \frac{{\partial \psi }}{{\partial x}}}}{{a_{{12}} \psi \pi ^{2} }}Fg \hfill \\ + \frac{1}{{a_{{12}} \pi }}vg' + \frac{{a_{{11}} }}{{a_{{12}} \pi }}g' = \frac{{a_{{12}} \frac{{\partial \psi }}{{\partial x}}\frac{{\partial \pi }}{{\partial x}}}}{{a_{{12}} \psi \pi ^{3} }}g'\eta + \frac{{a_{{12}} \left( {\frac{{\partial \pi }}{{\partial x}}} \right)^{2} }}{{a_{{12}} \pi ^{4} }}g''\eta ^{2} \hfill \\ + \frac{{a_{{12}} \frac{{\partial ^{2} \pi }}{{\partial x^{2} }}}}{{a_{{12}} \pi ^{3} }}g'\eta + \frac{{a_{{12}} \frac{{\partial ^{2} \psi }}{{\partial x^{2} }}}}{{a_{{12}} \psi \pi ^{2} }}g + \frac{{a_{{12}} \frac{{\partial \psi }}{{\partial x}}\frac{{\partial \pi }}{{\partial x}}}}{{a_{{12}} \psi \pi ^{3} }}g'\eta + g'' \hfill \\ \end{gathered}$$

46 $$\:\frac{\frac{{{\partial\:H}_{1}}_{w}}{\partial\:t}}{{\eta\:}_{0}{{H}_{1}}_{w}{\pi\:}^{2}}H+\frac{\frac{\partial\:\pi\:}{\partial\:t}}{{\eta\:}_{0}{\pi\:}^{3}}H{\prime\:}\eta\:+\frac{\frac{\partial\:{\Gamma\:}}{\partial\:t}}{{\eta\:}_{0}{\Gamma\:}{\pi\:}^{2}}H+\frac{\omega\:{u}_{w}\frac{{{\partial\:H}_{1}}_{w}}{\partial\:x}}{{\eta\:}_{0}{{H}_{1}}_{w}{\pi\:}^{2}}FH+\frac{\omega\:{u}_{w}\frac{\partial\:\pi\:}{\partial\:x}}{{\eta\:}_{0}{\pi\:}^{3}}FH{\prime\:}\eta\:+\frac{\omega\:{u}_{w}\frac{\partial\:{\Gamma\:}}{\partial\:x}}{{\eta\:}_{0}{\Gamma\:}{\pi\:}^{2}}FH+\frac{1}{{\eta\:}_{0}\pi\:}vH{\prime\:}=H{\prime\:}{\prime\:}$$

47 $$\:\frac{\frac{{{\partial\:H}_{2}}_{w}}{\partial\:t}}{{\eta\:}_{0}{{H}_{2}}_{w}{\pi\:}^{2}}J+\frac{\frac{\partial\:\pi\:}{\partial\:t}}{{\eta\:}_{0}{\pi\:}^{3}}J{\prime\:}\eta\:+\frac{\frac{\partial\:{\uplambda\:}}{\partial\:t}}{{\eta\:}_{0}{\uplambda\:}{\pi\:}^{2}}J+\frac{\omega\:{u}_{w}\frac{{{\partial\:H}_{2}}_{w}}{\partial\:x}}{{\eta\:}_{0}{{H}_{2}}_{w}{\pi\:}^{2}}FJ+\frac{\omega\:{u}_{w}\frac{\partial\:\pi\:}{\partial\:x}}{{\eta\:}_{0}{\pi\:}^{3}}FJ{\prime\:}\eta\:+\frac{\omega\:{u}_{w}\frac{\partial\:{\uplambda\:}}{\partial\:x}}{{\eta\:}_{0}{\uplambda\:}{\pi\:}^{2}}FJ+\frac{1}{{\eta\:}_{0}\pi\:}vJ{\prime\:}=J{\prime\:}{\prime\:}$$

48 $$\begin{gathered} \:\frac{{\frac{{\partial \:R_{w} }}{{\partial \:t}}}}{{D_{r} R_{w} \pi \:^{2} }}E + \frac{{\frac{{\partial \:\pi \:}}{{\partial \:t}}}}{{D_{r} \pi \:^{3} }}E\prime \:\eta \: + \frac{{\frac{{\partial \:\zeta \:}}{{\partial \:t}}}}{{D_{r} \zeta \:\pi \:^{2} }}E + \frac{{\omega \:u_{w} \frac{{\partial \:R_{w} }}{{\partial \:x}}}}{{D_{r} R_{w} \pi \:^{2} }}FE \hfill \\ + \frac{{\omega \:u_{w} \frac{{\partial \:\pi \:}}{{\partial \:x}}}}{{D_{r} \pi \:^{3} }}E\prime \:\eta \:F + \frac{{\omega \:u_{w} \frac{{\partial \:\zeta \:}}{{\partial \:x}}}}{{D_{r} \zeta \:\pi \:^{2} }}FE + \frac{1}{{D_{r} \pi \:}}vE\prime \: = \frac{{\frac{{\partial \:^{2} R_{w} }}{{\partial \:x^{2} }}}}{{R_{w} \pi \:^{2} }}E \hfill \\ + \frac{{\frac{{\partial \:R_{w} }}{{\partial \:x}}\frac{{\partial \:\pi \:}}{{\partial \:x}}}}{{R_{w} \pi \:^{3} }}E\prime \:\eta \: + \frac{{\frac{{\partial \:R_{w} }}{{\partial \:x}}\frac{{\partial \:\zeta \:}}{{\partial \:x}}}}{{R_{w} \zeta \:\pi \:^{2} }}E + \frac{{\frac{{\partial \:\zeta \:}}{{\partial \:x}}\frac{{\partial \:\pi \:}}{{\partial \:x}}}}{{\zeta \:\pi \:^{3} }}E\prime \:\eta \: \hfill \\ + \frac{{\left( {\frac{{\partial \:\pi \:}}{{\partial \:x}}} \right)^{2} }}{{\pi \:^{4} }}E\prime \:\prime \:\eta \:^{2} + \frac{{\frac{{\partial \:^{2} \pi \:}}{{\partial \:x^{2} }}}}{{\pi \:^{3} }}E\prime \:\eta \: + \frac{{\frac{{\partial \:^{2} \zeta \:}}{{\partial \:x^{2} }}}}{{\zeta \:\pi \:^{2} }}E + \frac{{\frac{{\partial \:\zeta \:}}{{\partial \:x}}\frac{{\partial \:\pi \:}}{{\partial \:x}}}}{{\zeta \:\pi \:^{3} }}E\prime \:\eta \: \hfill \\ + \frac{{\frac{{\partial \:\pi \:}}{{\partial \:x}}\frac{{\partial \:R_{w} }}{{\partial \:x}}}}{{R_{w} \pi \:^{3} }}E\prime \:\eta \: + \frac{{\frac{{\partial \:\zeta \:}}{{\partial \:x}}\frac{{\partial \:R_{w} }}{{\partial \:x}}}}{{R_{w} \zeta \:\pi \:^{2} }}E + E\prime \:\prime \: - \frac{{k_{r} c_{1} \Gamma \:H_{{1w}} }}{{D_{r} R_{w} \zeta \:\pi \:^{2} }}H + \frac{{k_{r} c_{2} \lambda \:H_{{2w}} }}{{D_{r} R_{w} \zeta \:\pi \:^{2} }}J \hfill \\ \end{gathered}$$

49 $$\begin{gathered} \:\frac{{\frac{{\partial \:P_{w} }}{{\partial \:t}}}}{{D_{p} P_{w} \pi \:^{2} }}S + \frac{{\frac{{\partial \:\pi \:}}{{\partial \:t}}}}{{D_{p} \pi \:^{3} }}\eta \:S\prime \: + \frac{{\frac{{\partial \:\varepsilon \:}}{{\partial \:t}}}}{{D_{p} \varepsilon \:\pi \:^{2} }}S + \frac{{\omega \:u_{w} \frac{{\partial \:P_{w} }}{{\partial \:x}}}}{{D_{p} P_{w} \pi \:^{2} }}FS \hfill \\ + \frac{{\omega \:u_{w} \frac{{\partial \:\pi \:}}{{\partial \:x}}}}{{D_{p} \pi \:^{3} }}\eta \:S\prime \:F + \frac{{\omega \:u_{w} \frac{{\partial \:\varepsilon \:}}{{\partial \:x}}}}{{D_{p} \varepsilon \:\pi \:^{2} }}FS + \frac{1}{{D_{p} \pi \:}}vS\prime \: = \frac{{\frac{{\partial \:^{2} P_{w} }}{{\partial \:x^{2} }}}}{{P_{w} \pi \:^{2} }}S \hfill \\ + \frac{{\frac{{\partial \:P_{w} }}{{\partial \:x}}\frac{{\partial \:\pi \:}}{{\partial \:x}}}}{{P_{w} \pi \:^{3} }}\eta \:S\prime \: + \frac{{\frac{{\partial \:P_{w} }}{{\partial \:x}}\frac{{\partial \:\smallint \:}}{{\partial \:x}}}}{{P_{w} \smallint \:\pi \:^{2} }}S + \frac{{\frac{{\partial \:\varepsilon }}{{\partial \:x}}\frac{{\partial \:\pi \:}}{{\partial \:x}}}}{{\varepsilon \:\pi \:^{3} }}\eta \:S\prime \: \hfill \\ + \frac{{\left( {\frac{{\partial \:\pi \:}}{{\partial \:x}}} \right)^{2} }}{{\pi \:^{4} }}\eta \:^{2} S\prime \:\prime \: + \frac{{\frac{{\partial \:^{2} \pi \:}}{{\partial \:x^{2} }}}}{{\pi \:^{3} }}\eta \:S\prime \: + \frac{{\frac{{\partial \:^{2} \varepsilon \:}}{{\partial \:x^{2} }}}}{{\varepsilon \:\pi \:^{2} }}S + \frac{{\frac{{\partial \:\smallint \:}}{{\partial \:x}}\frac{{\partial \:\pi \:}}{{\partial \:x}}}}{{\varepsilon \:\pi \:^{3} }}S\prime \:\prime \:\eta \: \hfill \\ + \frac{{\frac{{\partial \:\pi \:}}{{\partial \:x}}\frac{{\partial \:P_{w} }}{{\partial \:x}}}}{{P_{w} \pi \:^{3} }}S\prime \:\eta \: + \frac{{\frac{{\partial \:\smallint \:}}{{\partial \:x}}\frac{{\partial \:P_{w} }}{{\partial \:x}}}}{{P_{w} \varepsilon \:\pi \:^{2} }}S + S\prime \:\prime \: - \frac{{\alpha \:}}{{D_{p} \pi \:^{2} }}S + \frac{{\gamma \:\Delta T}}{{D_{p} P_{w} \varepsilon \:\pi \:^{2} }}\theta \: \hfill \\ + \frac{{\zeta \:\Delta \:C}}{{D_{p} P_{w} \varepsilon \:\pi \:^{2} }}C^{*} + \frac{{\delta \:\Gamma \:^{2} H_{{1w}} ^{2} }}{{D_{p} P_{w} \varepsilon \:\pi \:^{2} }}H^{2} + \frac{{\delta \:\lambda \:^{2} H_{{2w}} ^{2} }}{{D_{p} P_{w} \varepsilon \:\pi \:^{2} }}J^{2} \hfill \\ \end{gathered}$$

Where $\:\varDelta\:T={T}_{w}-{T}_{\infty\:},\varDelta\:C={C}_{w}-{C}_{\infty\:},\varDelta\:N={N}_{w}-{N}_{\infty\:}$. Derivatives with respect to $\:\eta\:$ are represented by dashes. It is necessary to adhere to the following relations in order to correctly convert Eqs. (41)–(49) into an ODE system:

50 $$\begin{gathered} \:A_{1} = \frac{{\omega \:\frac{{\partial \:u_{w} }}{{\partial \:x}}}}{{\pi \:}},A_{2} = \frac{{u_{w} \omega \:\frac{{\partial \:\pi \:}}{{\partial \:x}}}}{{\pi \:^{2} }},A_{3} = \frac{{u_{w} \frac{{\partial \:\omega \:}}{{\partial \:x}}}}{{\pi \:}},A_{4} = \frac{{\frac{{\partial \:u_{w} }}{{\partial \:t}}}}{{a_{1} u_{w} \pi \:^{2} }}, \hfill \\ A_{5} = \frac{{\frac{{\partial \:\pi \:}}{{\partial \:t}}}}{{a_{1} \pi \:^{3} }},A_{6} = \frac{{\frac{{\partial \:\omega \:}}{{\partial \:t}}}}{{a_{1} \omega \:\pi \:^{2} }},A_{7} = \frac{{\omega \:\frac{{\partial \:u_{w} }}{{\partial \:x}}}}{{a_{1} \pi \:^{2} }},A_{8} = \frac{{\omega \:u_{w} \frac{{\partial \:\pi \:}}{{\partial \:x}}}}{{a_{1} \pi \:^{3} }}, \hfill \\ A_{9} = \frac{{u_{w} \frac{{\partial \:\omega \:}}{{\partial \:x}}}}{{a_{1} \pi \:^{2} }},A_{{10}} = \frac{1}{{a_{1} \pi \:}},A_{{11}} = \frac{{a_{2} }}{{a_{1} \pi \:^{2} }},A_{{12}} = \frac{{a_{3} \Delta \:T}}{{a_{1} u_{w} \omega \:\pi \:^{2} }}, \hfill \\ A_{{13}} = \frac{{a_{4} \Delta \:C}}{{a_{1} u_{w} \omega \:\pi \:^{2} }},A_{{14}} = \frac{{a_{5} \Delta \:N\psi \:}}{{a_{1} u_{w} \omega \:\pi \:^{2} }},A_{{15}} = \frac{{a_{6} \lambda \:H_{{2w}} \Gamma \:\frac{{\partial \:H_{{1w}} }}{{\partial \:x}}}}{{a_{1} u_{w} \omega \:\pi \:^{2} }}, \hfill \\ A_{{16}} = \frac{{a_{6} \lambda \:H_{{2w}} H_{{1w}} \Gamma \:\frac{{\partial \:\pi \:}}{{\partial \:x}}}}{{a_{1} u_{w} \omega \:\pi \:^{3} }},A_{{17}} = \frac{{a_{6} \lambda \:H_{{2w}} H_{{1w}} \frac{{\partial \:\Gamma \:}}{{\partial \:x}}}}{{a_{1} u_{w} \omega \:\pi \:^{2} }}, \hfill \\ A_{{18}} = \frac{{a_{6} \lambda \:H_{{2w}} H_{{1w}} \Gamma \:}}{{a_{1} u_{w} \omega \:\pi \:}},A_{{19}} = \frac{{\frac{{\partial \:\pi \:}}{{\partial \:t}}}}{{a_{7} \pi \:^{3} }},A_{{20}} = \frac{{\omega \:u_{w} \frac{{\partial \:\pi \:}}{{\partial \:x}}}}{{a_{7} \pi \:^{3} }}, \hfill \\ A_{{21}} = \frac{1}{{a_{7} \pi \:}},A_{{22}} = \frac{{a_{8} \Delta \:C}}{{a_{7} }},A_{{23}} = \frac{{a_{9} \Delta \:T}}{{a_{7} }},A_{{24}} = \frac{{a_{{10}} }}{{a_{7} \pi \:^{2} }} \hfill \\ \end{gathered}$$

Likewise, the formulae, (41) through (49), admit the following functions to ensure the transformation into a system of ODEs.

51 $$\begin{gathered} \:\pi \: = c_{1} ,u_{w} = c_{2} e^{{c_{1}^{2} t}} ,\omega \: = c_{3} e^{{ - c_{1}^{2} t}} ,\psi \: = c_{4} ,H_{{1w}} = c_{5} e^{{c_{1}^{2} t}} ,\Gamma \: = c_{6} e^{{ - c_{1}^{2} t}} , \hfill \\ H_{{2w}} = c_{7} e^{{c_{1}^{2} t}} ,\lambda \: = c_{8} e^{{ - c_{1}^{2} t}} ,R_{w} = c_{9} e^{{c_{1}^{2} t}} ,\zeta \: = c_{{10}} e^{{ - c_{1}^{2} t}} ,P_{w} = c_{{11}} e^{{c_{1}^{2} t}} ,\varepsilon \: = c_{{12}} e^{{ - c_{1}^{2} t}} \hfill \\ \end{gathered}$$

The following displays the ODE system in its ultimate form:

52 $$\:{v}^{{\prime\:}}=0,$$

53 $$\:{F}^{{\prime\:}{\prime\:}}-\frac{{a}_{2}}{{a}_{1}{c}_{1}^{2}}F+\frac{{a}_{3}\varDelta\:T}{{a}_{1}{c}_{2}{c}_{3}{c}_{1}^{2}}\theta\:-\frac{{a}_{4}\varDelta\:C}{{a}_{1}{c}_{2}{c}_{3}{c}_{1}^{2}}{C}^{*}-\frac{{a}_{5}\varDelta\:N{c}_{4}}{{a}_{1}{c}_{2}{c}_{3}{c}_{1}^{2}}g+\frac{{a}_{6}{c}_{7}{c}_{8}{c}_{5}{c}_{6}}{{a}_{1}{c}_{2}{c}_{3}{c}_{1}}J{H}^{{\prime\:}}-\frac{1}{{a}_{1}{c}_{1}}v{F}^{{\prime\:}}=0$$

54 $$\:{\theta\:}^{{\prime\:}{\prime\:}}+\frac{{a}_{8}{\Delta\:}C}{{a}_{7}}{c}^{{*}^{{\prime\:}}}{\theta\:}^{{\prime\:}}+\frac{{a}_{9}{\Delta\:}T}{{a}_{7}}{{\theta\:}^{{\prime\:}}}^{2}+\frac{{a}_{10}}{{a}_{7}{c}_{1}^{2}}\theta\:-\frac{1}{{a}_{7}{c}_{1}}v{\theta\:}^{{\prime\:}}=0$$

55 $$\:{{C}^{*}}^{{\prime\:}{\prime\:}}-\frac{1}{{D}_{B}{c}_{1}}v{{C}^{*}}^{{\prime\:}}=0$$

56 $$\:{g}^{{\prime\:}{\prime\:}}-\frac{1}{{a}_{12}{c}_{1}}v{g}^{{\prime\:}}-\frac{{a}_{11}}{{a}_{12}{c}_{1}}{g}^{{\prime\:}}=0$$

57 $$\:{H}^{{\prime\:}{\prime\:}}-\frac{1}{{\eta\:}_{0}{c}_{1}}v{H}^{{\prime\:}}=0$$

58 $$\:{J}^{{\prime\:}{\prime\:}}-\frac{1}{{\eta\:}_{0}{c}_{1}}v{J}^{{\prime\:}}=0$$

59 $$\:{E}^{{\prime\:}{\prime\:}}-\frac{{k}_{r}{{c}_{1}}^{*}{c}_{5}{c}_{6}}{{D}_{r}{c}_{9}{c}_{10}{c}_{1}^{2}}H+\frac{{k}_{r}{{c}_{2}}^{*}{c}_{7}{c}_{8}}{{D}_{r}{c}_{9}{c}_{10}{c}_{1}^{2}}J-\frac{1}{{D}_{r}{c}_{1}}v{E}^{{\prime\:}}=0$$

60 $$\:{S}^{{\prime\:}{\prime\:}}-\frac{\alpha\:}{{D}_{p}{c}_{1}^{2}}S+\frac{\gamma\:\varDelta\:T}{{D}_{p}{c}_{11}{c}_{12}{c}_{1}^{2}}\theta\:+\frac{{\zeta\:}^{*}\varDelta\:C}{{D}_{p}{c}_{11}{c}_{12}{c}_{1}^{2}}{C}^{*}+\frac{\delta\:{c}_{5}^{2}{c}_{6}^{2}}{{D}_{p}{c}_{11}{c}_{12}{c}_{1}^{2}}{H}^{2}+\frac{\delta\:{c}_{7}^{2}{c}_{8}^{2}}{{D}_{p}{c}_{11}{c}_{12}{c}_{1}^{2}}{J}^{2}-\frac{1}{{D}_{p}{c}_{1}}v{S}^{{\prime\:}}=0$$

The corresponding boundary conditions become:

$$\:\eta\:=0$$

$$\:F=1,\:v={v}_{0},\:H=1,\:J=1,E=1,S=1,\theta\:=1,\:\:{C}^{*}=1,{\:\:N}^{*}=1.$$

$$\:\eta\:\to\:\infty\:$$

61 $$\:F=0,\:H=0,\:J=0,E=0,S=0,\theta\:=0,\:\:{C}^{*}=0,{\:\:N}^{*}=0.\:\:\:$$

Moreover, the nanofluid parameters are obtained using the following relationships.

62 $$\:{\mu\:}_{hnf}=\frac{{\mu\:}_{f}}{{\left(1-{\varphi\:}_{n1}\right)}^{2.5}{\left(1-{\varphi\:}_{n2}\right)}^{2.5}{\left(1-{\varphi\:}_{n3}\right)}^{2.5}}$$

63 $$\:{\rho\:}_{hnf}={\rho\:}_{f}\left[\left(1-{\varphi\:}_{n1}\right)\left(\left(1-{\varphi\:}_{n2}\right)\left[\left(1-{\varphi\:}_{n3}\right){\rho\:}_{f}+{\varphi\:}_{n3}{\rho\:}_{n3}\right]+{\varphi\:}_{n2}{\rho\:}_{n2}\right)+{\varphi\:}_{n1}{\rho\:}_{n1}\right]$$

64 $$\:{\left(\rho\:{c}_{p}\right)}_{hnf}={\left(\rho\:{c}_{p}\right)}_{f}\left[\left(1-{\varphi\:}_{n1}\right)\left(\left(1-{\varphi\:}_{n2}\right)\left[\left(1-{\varphi\:}_{n3}\right){\left(\rho\:{c}_{p}\right)}_{f}+{\varphi\:}_{n3}{\left(\rho\:{c}_{p}\right)}_{3}\right]+{\varphi\:}_{n2}{\left(\rho\:{c}_{p}\right)}_{2}\right)+{\varphi\:}_{n1}{\left(\rho\:{c}_{p}\right)}_{1}\right]$$

65 $$\begin{gathered} \:k_{{hnf}} = k_{f} \left( {\frac{{k_{{n1}} + \left( {n - 1} \right)k_{f} - \left( {n - 1} \right)\varphi \:_{{n1}} \left( {k_{f} - k_{{n1}} } \right)}}{{k_{{n1}} + \left( {n - 1} \right)k_{f} + \varphi \:_{{n1}} \left( {k_{f} - k_{{n1}} } \right)}}} \right) \hfill \\ \left( {\frac{{k_{{n2}} + \left( {n - 1} \right)k_{f} - \left( {n - 1} \right)\varphi \:_{{n2}} \left( {k_{f} - k_{{n2}} } \right)}}{{k_{{n2}} + \left( {n - 1} \right)k_{f} + \varphi \:_{{n2}} \left( {k_{f} - k_{{n2}} } \right)}}} \right) \hfill \\ \left( {\frac{{k_{{n3}} + \left( {n - 1} \right)k_{f} - \left( {n - 1} \right)\varphi \:_{{n3}} \left( {k_{f} - k_{{n3}} } \right)}}{{k_{{n3}} + \left( {n - 1} \right)k_{f} + \varphi \:_{{n3}} \left( {k_{f} - k_{{n3}} } \right)}}} \right) \hfill \\ \end{gathered}$$

## Result and discussion

### Validation of the results

**Fig. 2:**
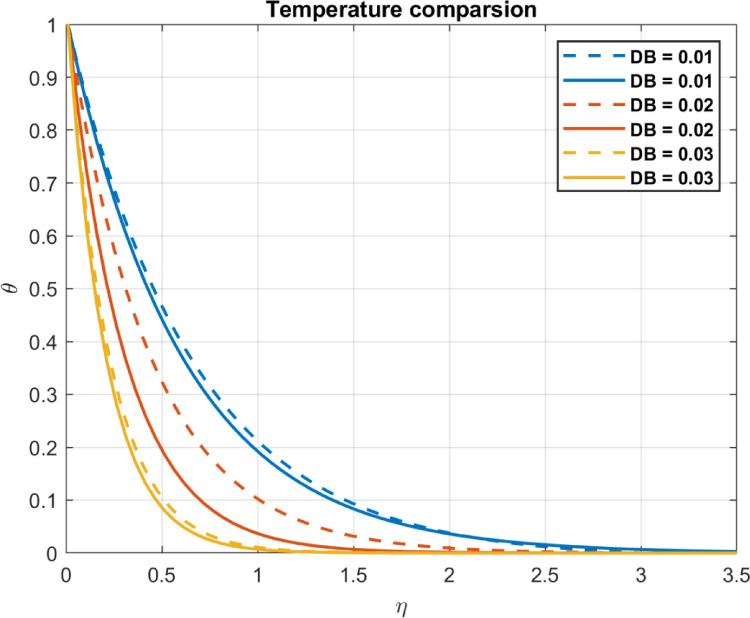
Comparison of the Brownian motion coefficient $\:DB$ with temperature; solid for Rashed et al.^57^, dashed lines for our research.

**Fig. 3:**
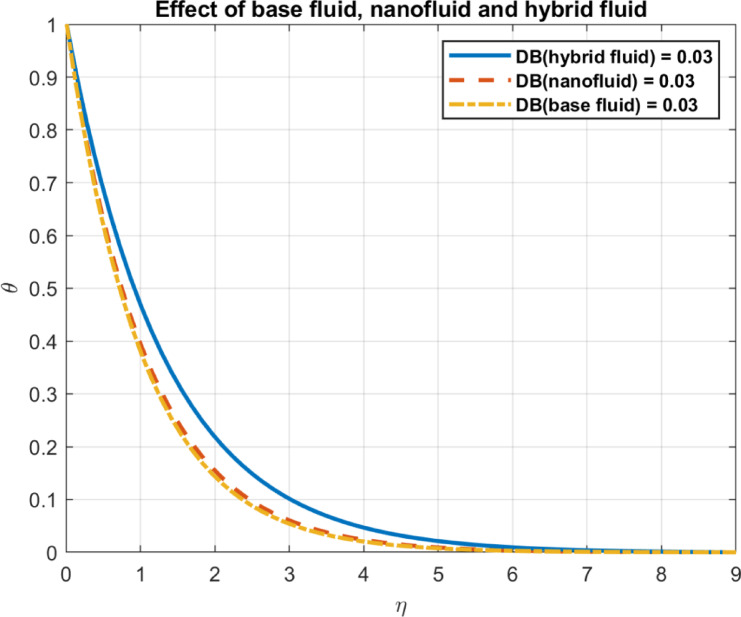
The effect of different number of nanoparticles.

Results are partially verified against the findings of Rashed et al.^57^ showing consistency in Brownian motion trends. The Brownian motion coefficient ($\:{D}_{B}$) is utilized as a benchmark for comparison, demonstrating a high degree of agreement, as depicted in Fig. 2. Furthermore, the effects of incorporating base fluid without nanoparticles, nanofluid with only one type of nanoparticles, and hybrid nanofluid incorporating two types of nanomaterials are illustrated in Fig. 3. The results emphasize the effectiveness of using hybrid mix instead of only one type of nanomaterials. Also, compare the outcomes with some experimental works show in Table 1.

**Table 1:** Comparison between the outcomes with some experimental works.

Parameter/Phenomenon	Manuscript finding	Experimental work	Comparison outcome
Bioconvection ($$Pe,D_{n} )$$	Higher $$\:\varvec{P}\varvec{e}$$ and $$\:{\varvec{D}}_{\varvec{n}}$$ promote uniform bacterial distribution and reduce density gradients (Figs. 14, 15, 21 and 22).	Pedley et al.^58^, confirmed that microbial motility leads to enhanced mixing and reduced density gradients	Qualitative agreement on mixing and density trends; quantitative comparison limited by differing setups.
Nanoparticle transport $(D_{B} ,S_{t} )$	Higher $\:{\varvec{D}}_{\varvec{B}}$ increases temperature but reduces heat flux; $\:{\varvec{S}}_{\varvec{t}}$ reduces temperature and heat flux (Figs. 4, 5, 6, 10, 11 and 12).	Wen et al.^59^, Brownian motion enhances thermal conductivity; Haddad et al.^60^: Thermophoresis boosts heat transfer.	Partial agreement on $\:{\varvec{D}}_{\varvec{B}}$​; discrepancy on $\:{\varvec{S}}_{\varvec{t}}\:$due to bioconvection effects; quantitative comparison limited.
Magnetic effects $\left( {M,\eta _{0} } \right)$	Higher $\:\varvec{M}$ reduces velocity; higher $\:{\varvec{\eta\:}}_{0}$promotes uniform magnetic fields (Figs. 13, 23, 24, 25 and 26).	Odenbach^61^: Magnetic fields reduce velocity; Rosensweig^62^: Diffusivity enhances field penetration.	Strong qualitative and trend-based agreement; quantitative comparison limited by unspecified parameters.
Pollutant removal $\left( {\alpha ,D_{p} } \right)$	Higher $\:\varvec{\alpha\:}$ and $\:{D}_{p}$ enhance pollutant removal and dispersion (Figs. 17, 18 and 19).	Yao et al.^63^: Magnetic fields improve removal; A. Okubo and S. A. Levin^64^: Diffusion enhances adsorption.	Qualitative agreement on removal efficiency; specific pollutant types and efficiencies differ.

**Fig. 4:**
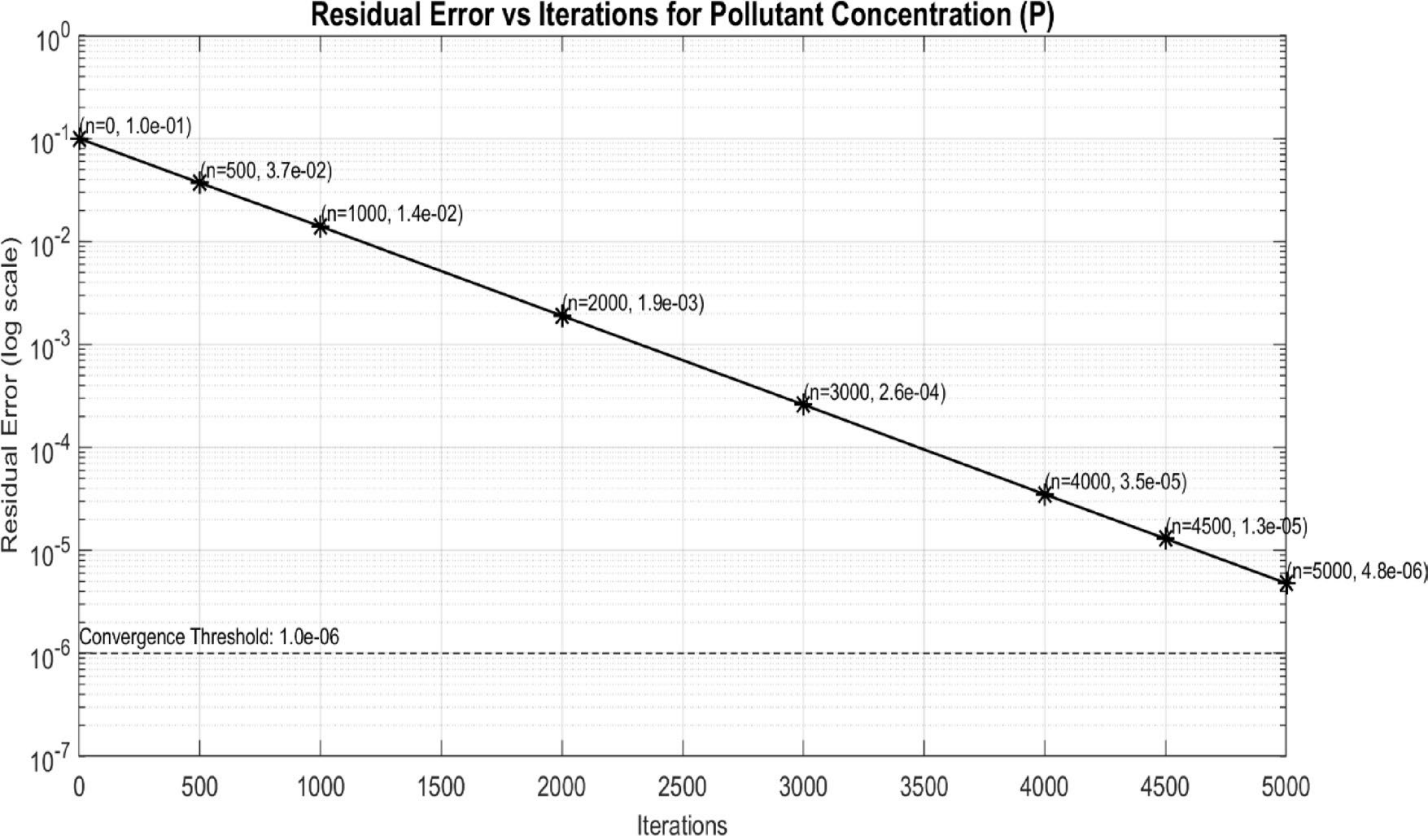
Residual error vs. iterations for pollutant concentration (p).

**Fig. 5:**
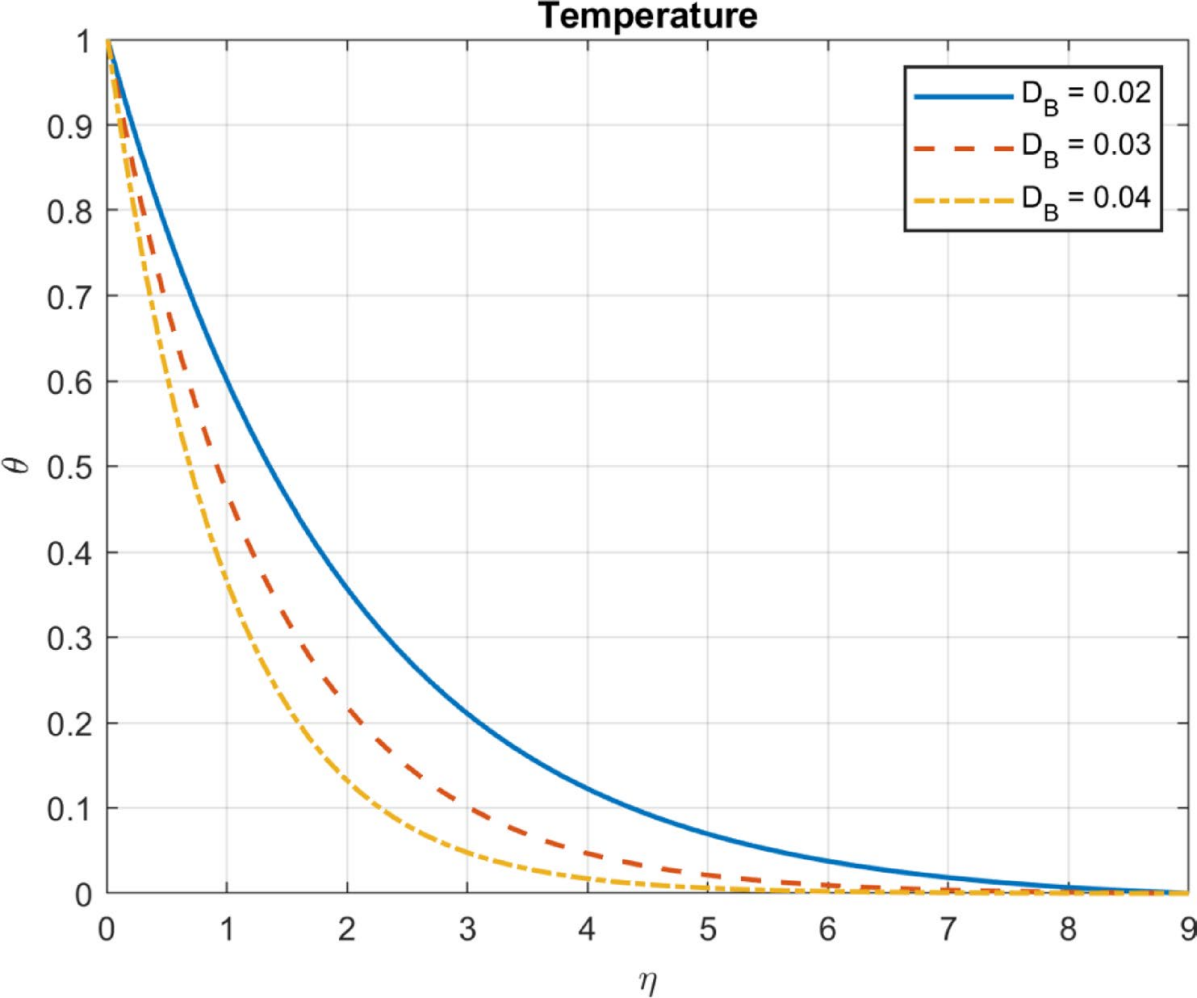
The influence of D_*B*_ on temperature.

**Fig. 6:**
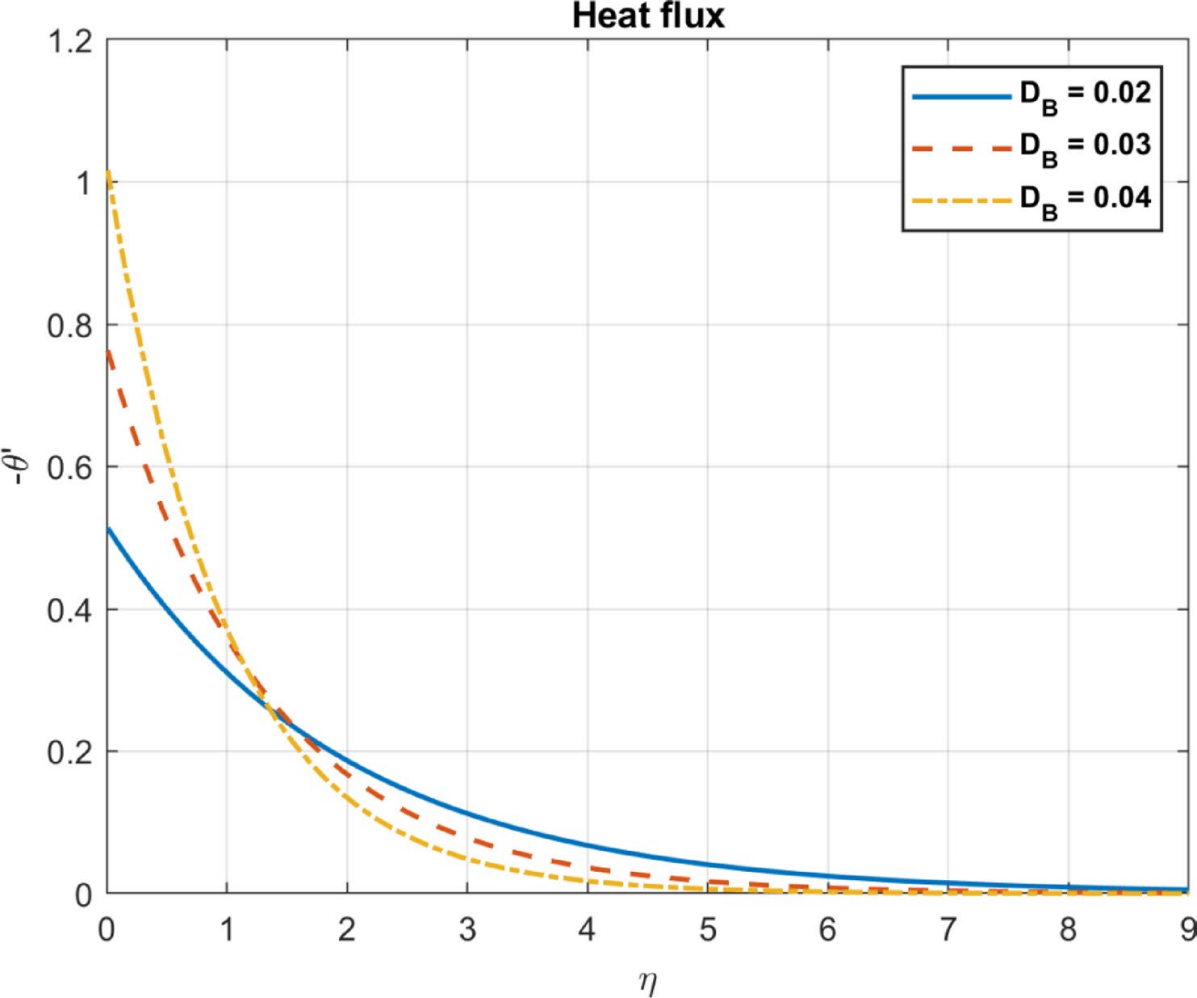
The influence of D_*B*_ on heat flux.

**Fig. 7:**
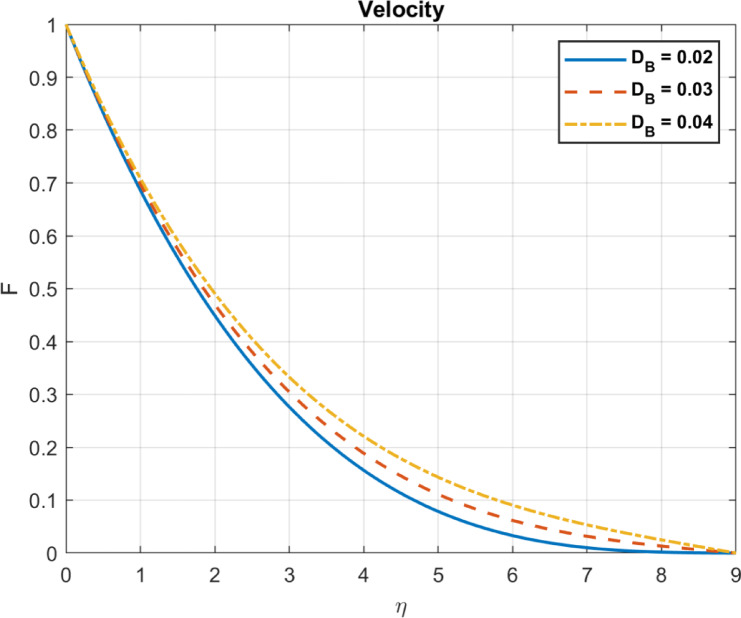
The influence of D_*B*_ on velocity.

**Fig. 8:**
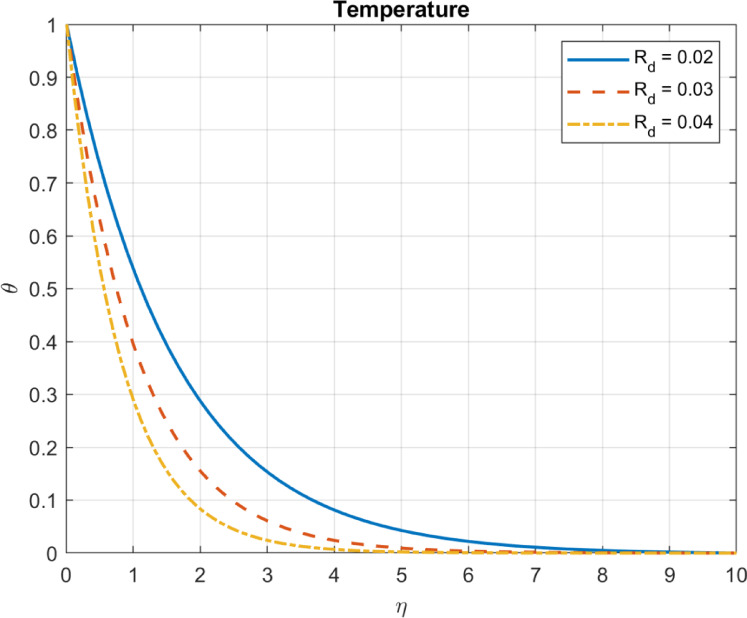
The influence of $\:{R}_{d}$ on temperature.

**Fig. 9:**
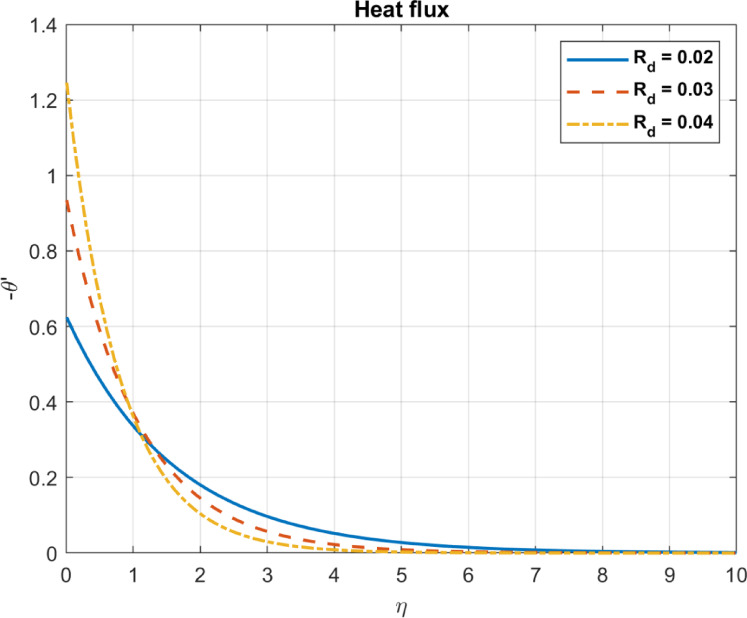
The influence of $\:{R}_{d}$ on heat flux.

**Fig. 10:**
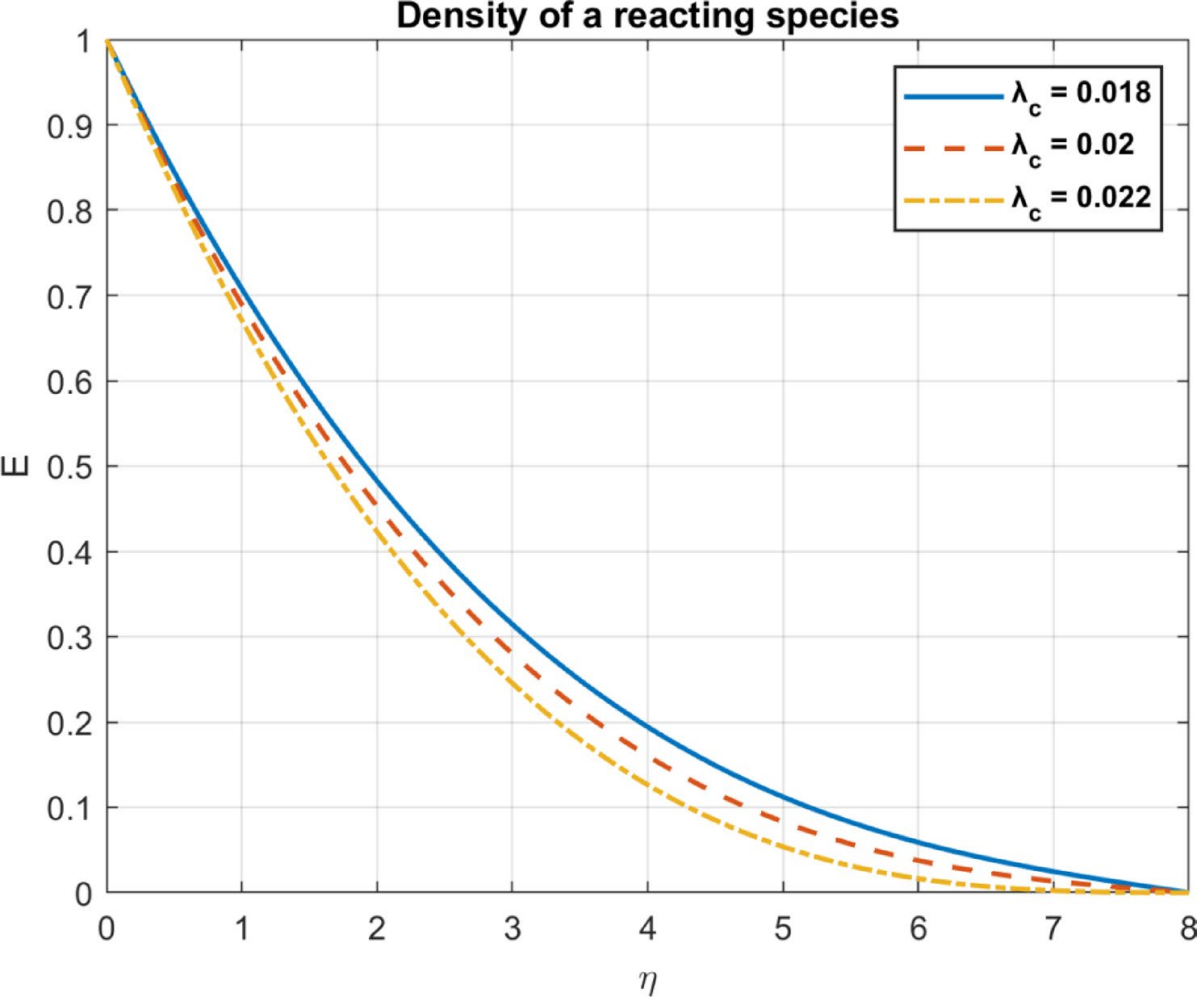
The influence of $\:{\lambda\:}_{c}$ on density of a reacting species.

**Fig. 11:**
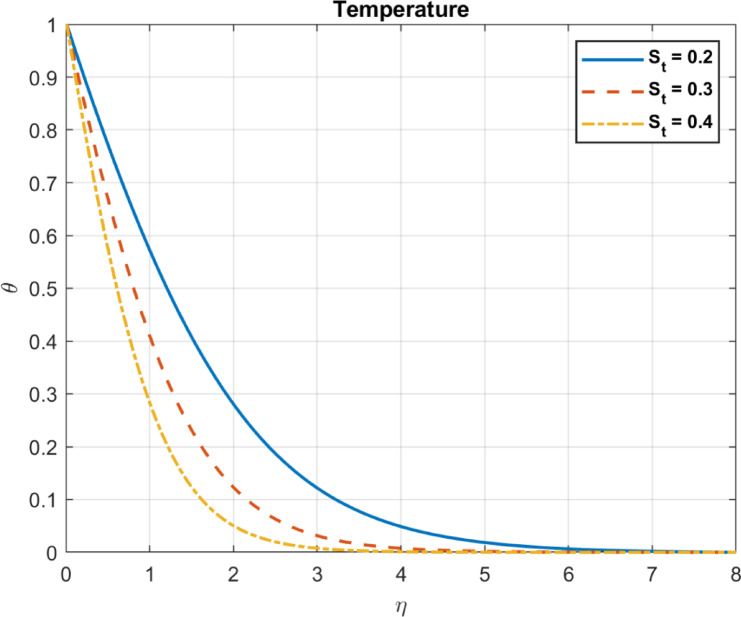
The influence of *S*_*t*_ on temperature.

**Fig. 12:**
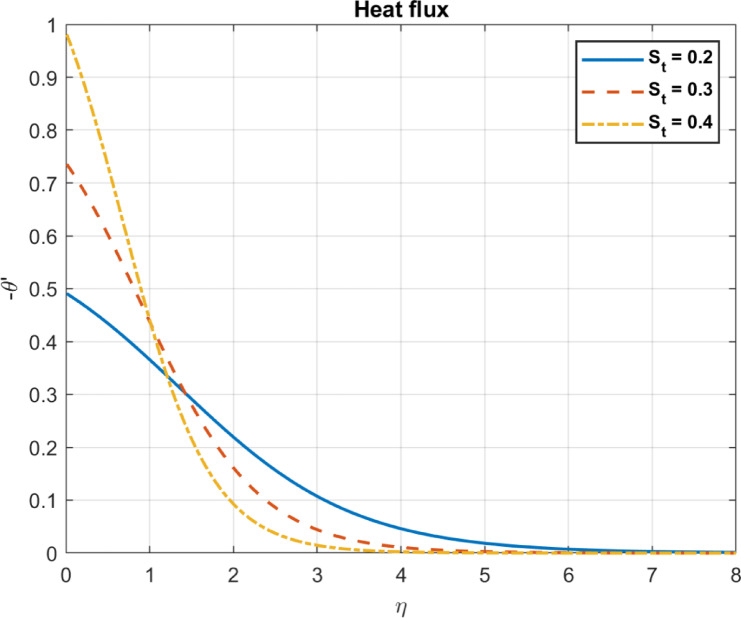
The influence of $\:{S}_{t}$ on heat flux.

**Fig. 13:**
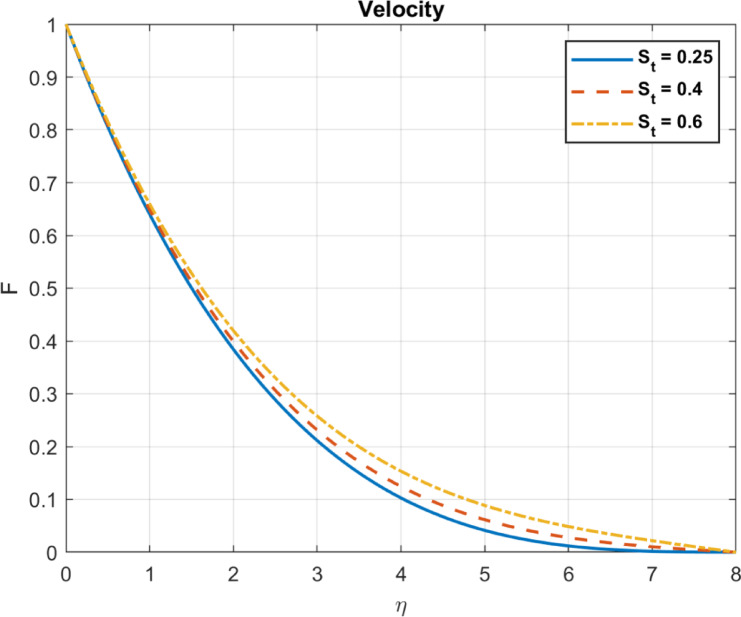
The influence of $\:{S}_{t}$ on velocity.

**Fig. 14:**
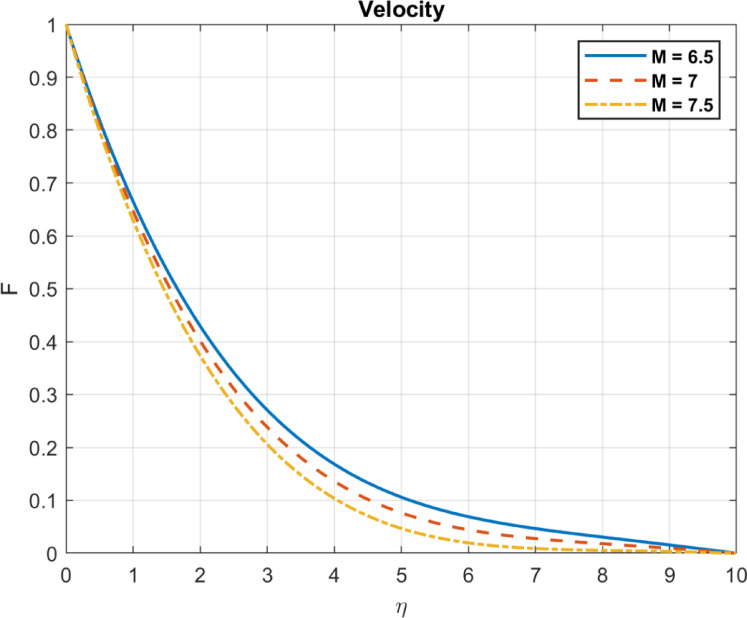
The influence of $\:M$ on velocity.

**Fig. 15:**
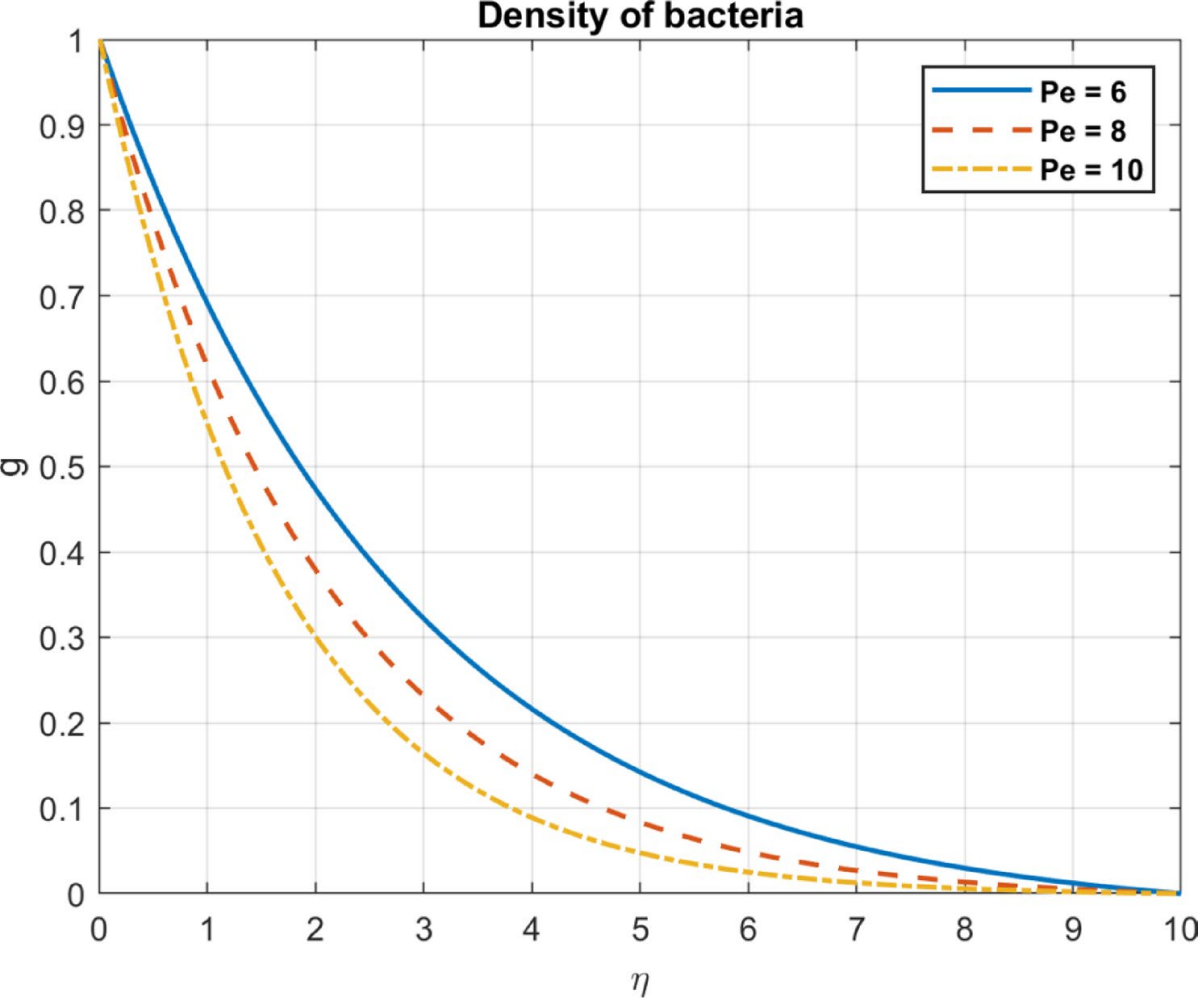
The influence of *P*_*e*_ on density of bacteria.

**Fig. 16:**
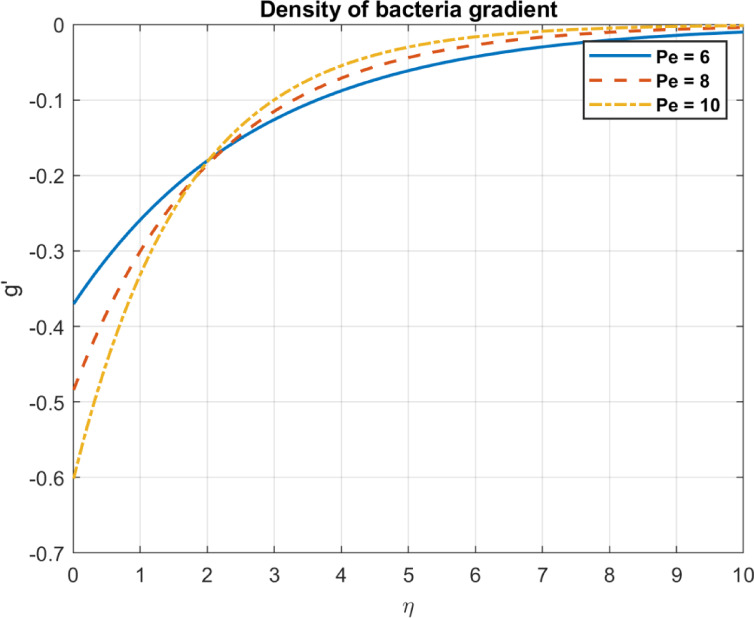
The influence of $\:Pe$ on density of bacteria gradient.

**Fig. 17:**
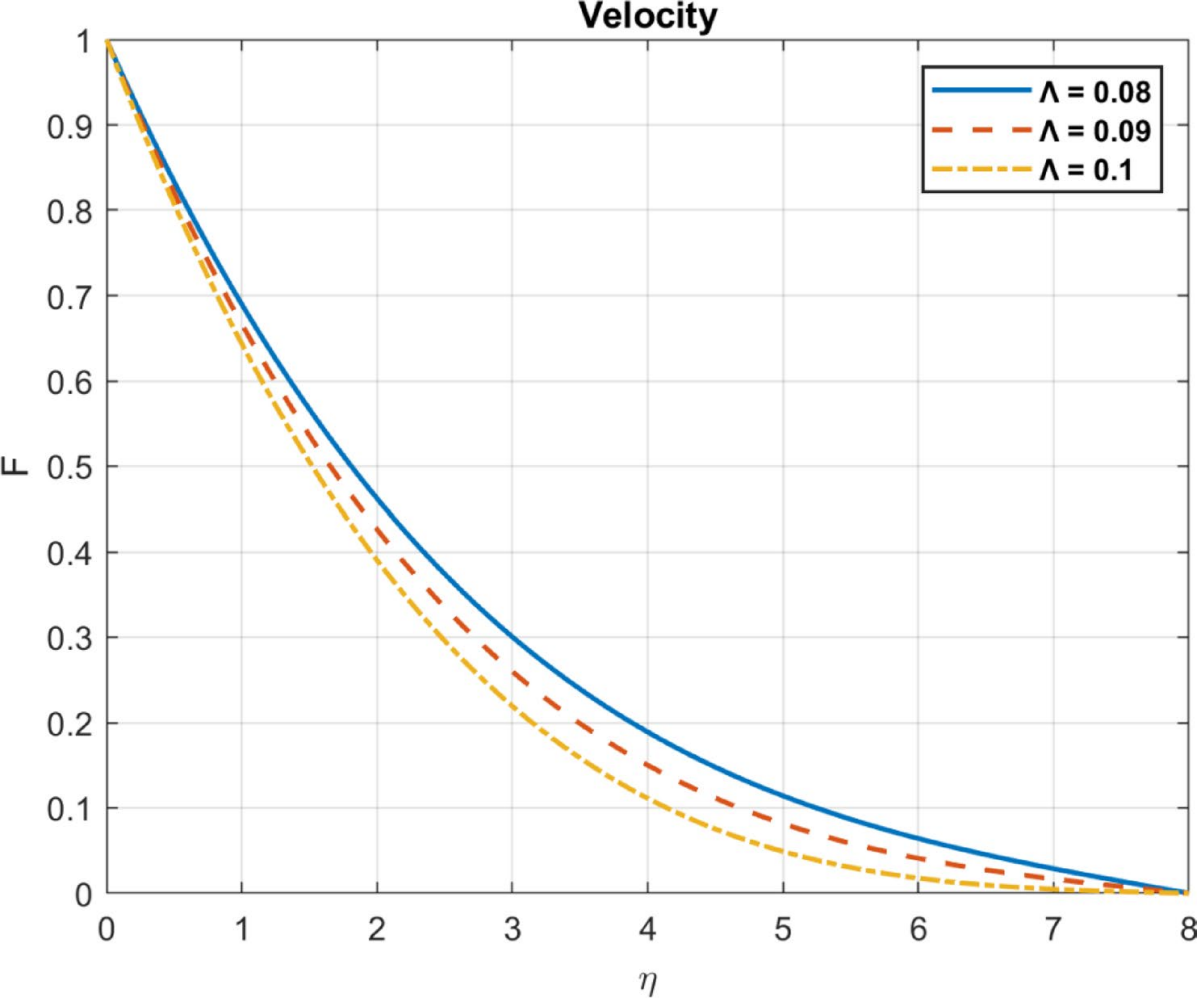
The influence of $\:\varLambda\:$ on velocity.

**Fig. 18:**
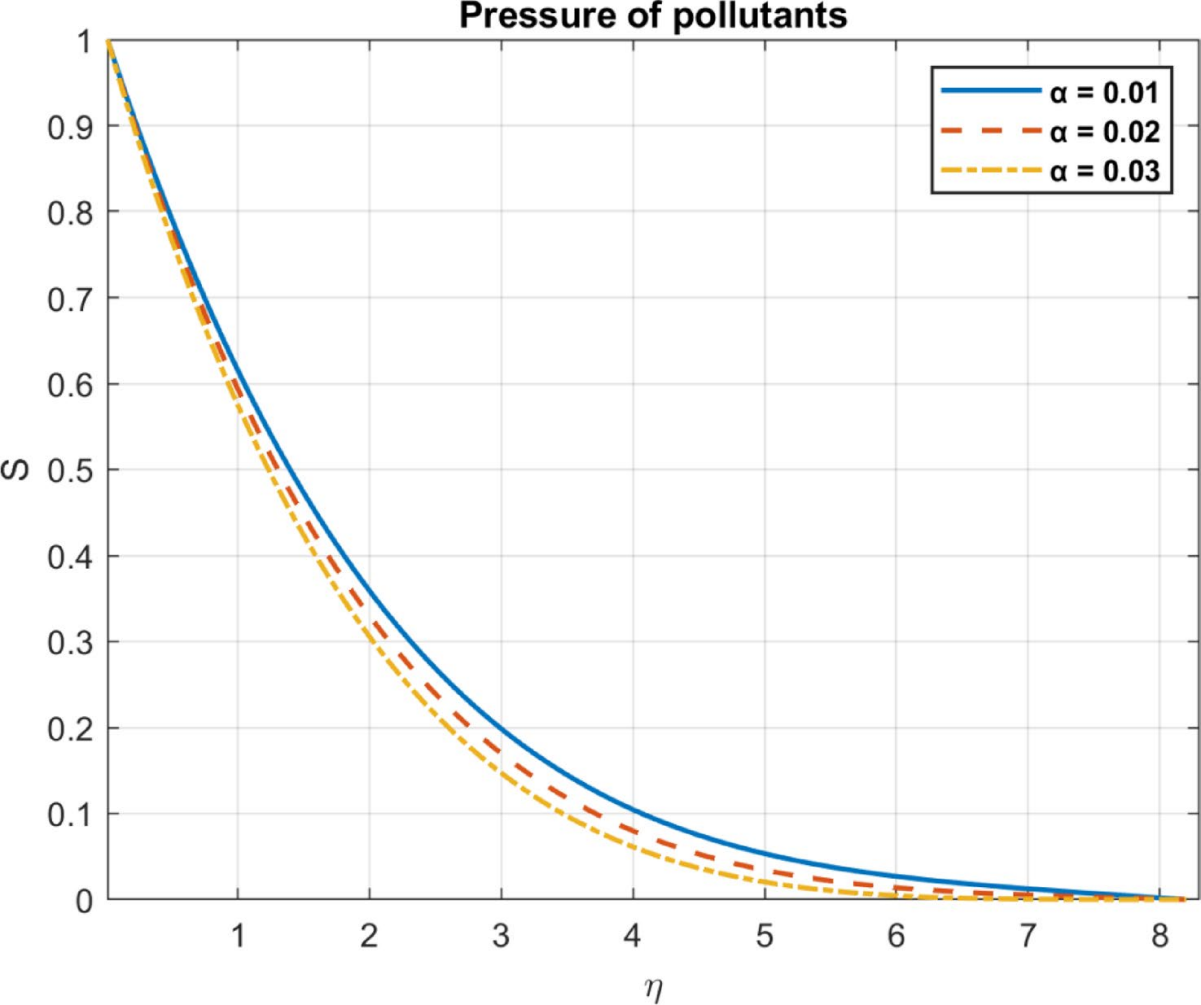
The influence of *α* on pressure of pollutants.

**Fig. 19:**
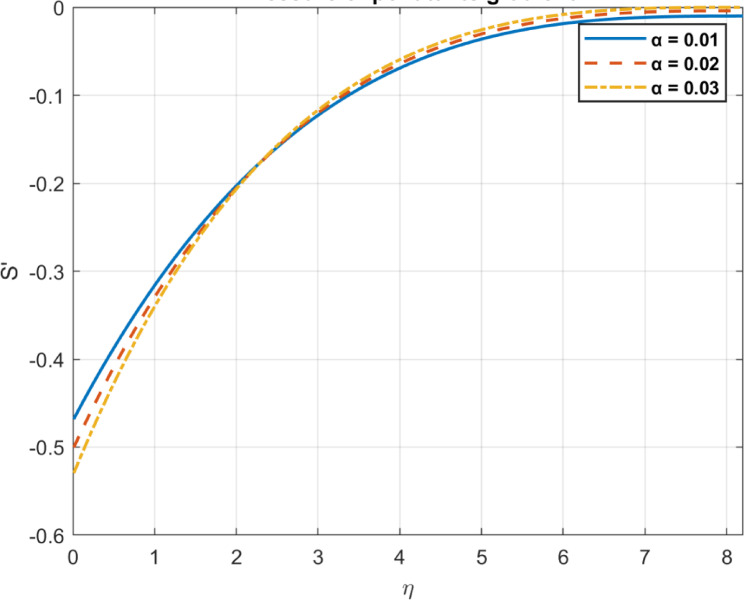
The influence of $\:\alpha\:$ on pressure gradient of pollutants.

**Fig. 20:**
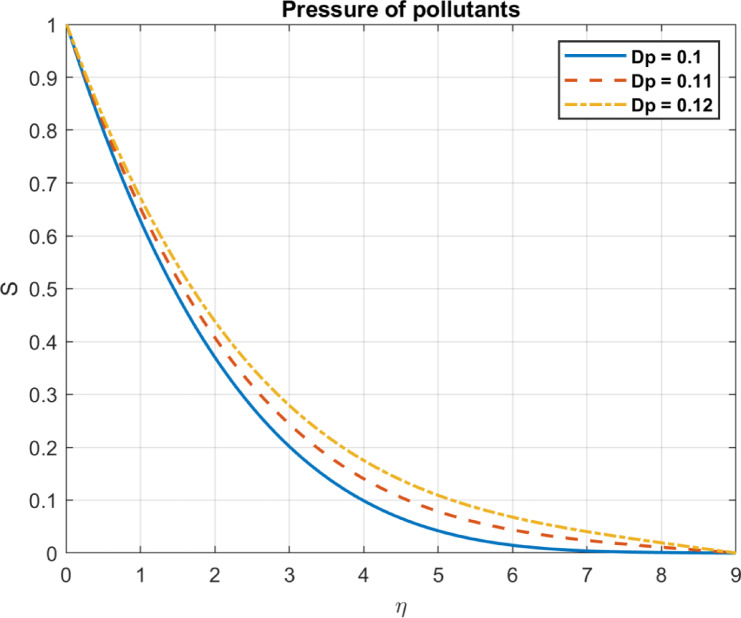
The influence of $\:Dp$ on pressure of pollutants.

**Fig. 21:**
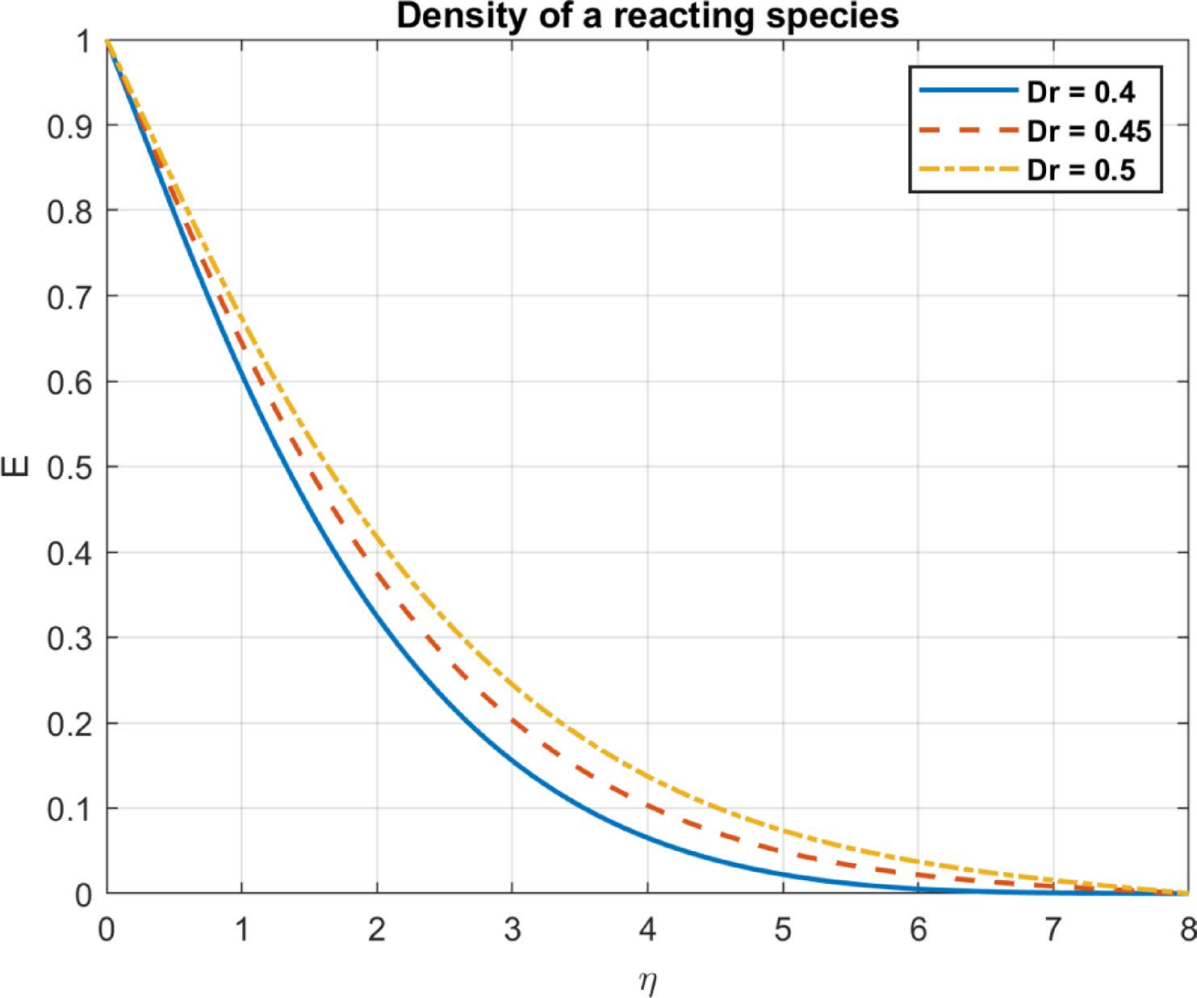
The influence of $\:{D}_{r}$ on density of a reacting species.

### Convergence and error analysis

The convergence of the method and the error analysis are depicted hereafter. A reference step value, $\:{h}_{ref}=0.001\:and\:0.0001$, is introduce to be compared to other three step values, $\:h=0.1,\:0.05,\:and\:0.025$. The global error for each variable in the study is evaluated and tabulated in the following tables. Table 2 depicts the system variables at $\:\eta\:=0.5,\:{h}_{ref}=0.001$, while Table 3 illustrates the global errors. Smaller step values result in better fitting and smaller errors.

**Table 2:** The system variables at different step values, and $\:{\text{h}}_{\text{r}\text{e}\text{f}}=0.001$.

$$\eta ~ \in \left[ {0,1} \right]$$				
Using $\:\eta\:=0.5$				
	$\:{h}_{ref}=0.001$	$\:{h}_{1}=0.1$	$\:{h}_{2}=0.05$	$\:{h}_{3}=0.025$
Y1	0.7322	0.8555	0.7559	0.7455
Y2	−0.2640	−0.2508	−0.5152	−0.5161
Y3	0.4828	0.5000	0.5358	0.5361
Y4	−0.6647	−0.6881	−0.7374	−0.7145
Y5	0.2960	0.1920	0.1904	0.1824
Y6	−0.2822	−0.3562	−0.3062	−0.3610
Y7	0.7123	0.7102	0.7003	0.6821
Y8	−0.4997	−0.4983	−0.5046	−0.5221
Y9	0.8367	0.8684	0.8477	0.8887
Y10	−0.1791	−0.2254	−0.2939	−0.2028
Y11	0.8457	0.8903	0.8892	0.8957
Y12	−0.2704	−0.1934	−0.1959	−0.1938
Y13	0.9210	0.9721	0.9688	0.9211
Y14	−0.3096	−0.4198	−0.4947	−0.3508
Y15	0.5624	0.6020	0.5771	0.5611
Y16	−0.3265	−0.4815	−0.5000	−0.5200

**Table 3:** The global error of the system’s variable.

$$\eta ~ \in \left[ {0,1} \right]$$			
Using $\:\:\eta\:=0.5$			
	$\:{h}_{1}=0.1$	$\:{h}_{2}=0.05$	$\:{h}_{3}=0.025$
Error in y1	0.1233	0.0237	0.0133
Error in y2	0.0132	0.2512	0.2521
Error in y3	0.0172	0.0530	0.0533
Error in y4	0.0234	0.0727	0.0498
Error in y5	0.1040	0.1056	0.1136
Error in y6	0.0740	0.0240	0.0788
Error in y7	0.0021	0.0120	0.0302
Error in y8	0.0014	0.0049	0.0224
Error in y9	0.0317	0.0110	0.0520
Error in y10	0.0461	0.1148	0.0237
Error in y11	0.0446	0.0435	0.0500
Error in y12	0.0770	0.0745	0.0766
Error in y13	0.0511	0.0478	0.0001
Error in y14	0.1102	0.1851	0.0412
Error in y15	0.0396	0.0147	0.0013
Error in y16	0.1550	0.1735	0.1935

The Fig. 4 shows a plot of residual error (on a log scale) versus iterations for pollutant concentration (P), with a convergence threshold of 1.0e-6. The data points indicate the residual error decreasing as iterations increase.

The residual error steadily decreases and falls below the specified convergence threshold of 1.0 × 10^{-6} after 5000 iterations, reaching a value of 4.8 × 10^{-6}. Sequel to Li et al.^65^ and Wang et al.^66^, this behavior confirms that the system has achieved convergence based on the predefined criterion. The consistent decline in error indicates numerical stability and reliability of the iterative solver. Moreover, the convergence pattern suggests that the selected discretization and solver settings are well-suited for the problem configuration.

### Brownian motion coefficient, $\:{\text{D}}_{B}$

The Brownian motion coefficient, or $\:{D}_{B}$, measures the rate at which particles disperse in a fluid due to random collisions with its molecules. Several factors, such as thermal conductivity, specific heat capacity, and external energy input, influence temperature distribution in a fluid system. In some chemical reactions or processes involving nanoparticles, Enhanced Brownian motion can increase reaction rates, potentially leading to localized heating due to exothermic reactions. Nanoparticles with increased Brownian motion can scatter throughout the fluid more readily. Increased Brownian motion enhances heat transfer by promoting energy transport through nanoparticle movement. The diffusion rate of nanoparticles increases with DB due to more frequent molecular collisions. A higher DB enhances heat transfer due to increased nanoparticle mobility, leading to elevated temperatures and heat flux, as plotted in Figs. 5, 6 and 7.

**Fig. 22:**
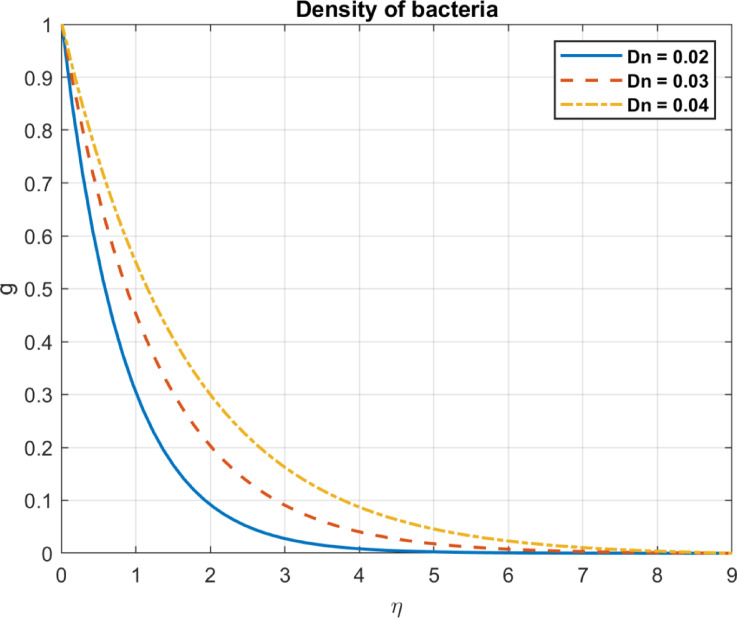
The influence of *D*_*n*_ on density of bacteria.

### Thermal radiation parameter ($\:{R}_{d}=\:\left(4{\upsigma\:}\:{\text{T}}_{\infty\:}^{3}\right)\:/\:{\text{k}}_{hnf}$)

The thermal radiation parameter ($\:{R}_{d}$) quantifies the relative contribution of radiative heat transfer in a system. It assesses the relative significance of radiative heat transfer over conduction and convection. As $\:{R}_{d}$ increases, i.e., thermal radiation becomes more significant over conduction and convection, the following influences are seen in a bioconvection system:

As the thermal radiation parameter $\:{R}_{d}$ increases, more energy is introduced into the system through radiative heat transfer, potentially raising the fluid temperature, especially in optically thick systems where radiation is absorbed Fig. 8 may reflect specific model assumptions (e.g., boundary conditions or optically thin systems). A higher $\:{R}_{d}$ implies stronger radiative heat transfer effects relative to conduction and convection in the modeled system. However, as radiative transfer increases, the relative contribution of conduction and convection may decrease, but total heat flux may still rise, as energy is primarily transferred through radiation. These trends are consistent with the expected behavior of systems with increasing $\:{R}_{d}$, as shown in Figs. 8 and 9.

**Fig. 23:**
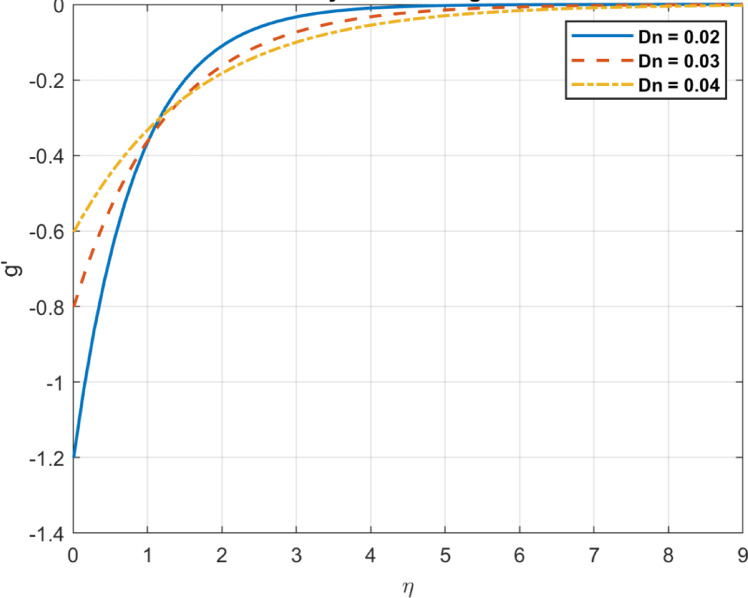
The influence of $\:{D}_{n}$ on density gradient of bacteria.

**Fig. 24:**
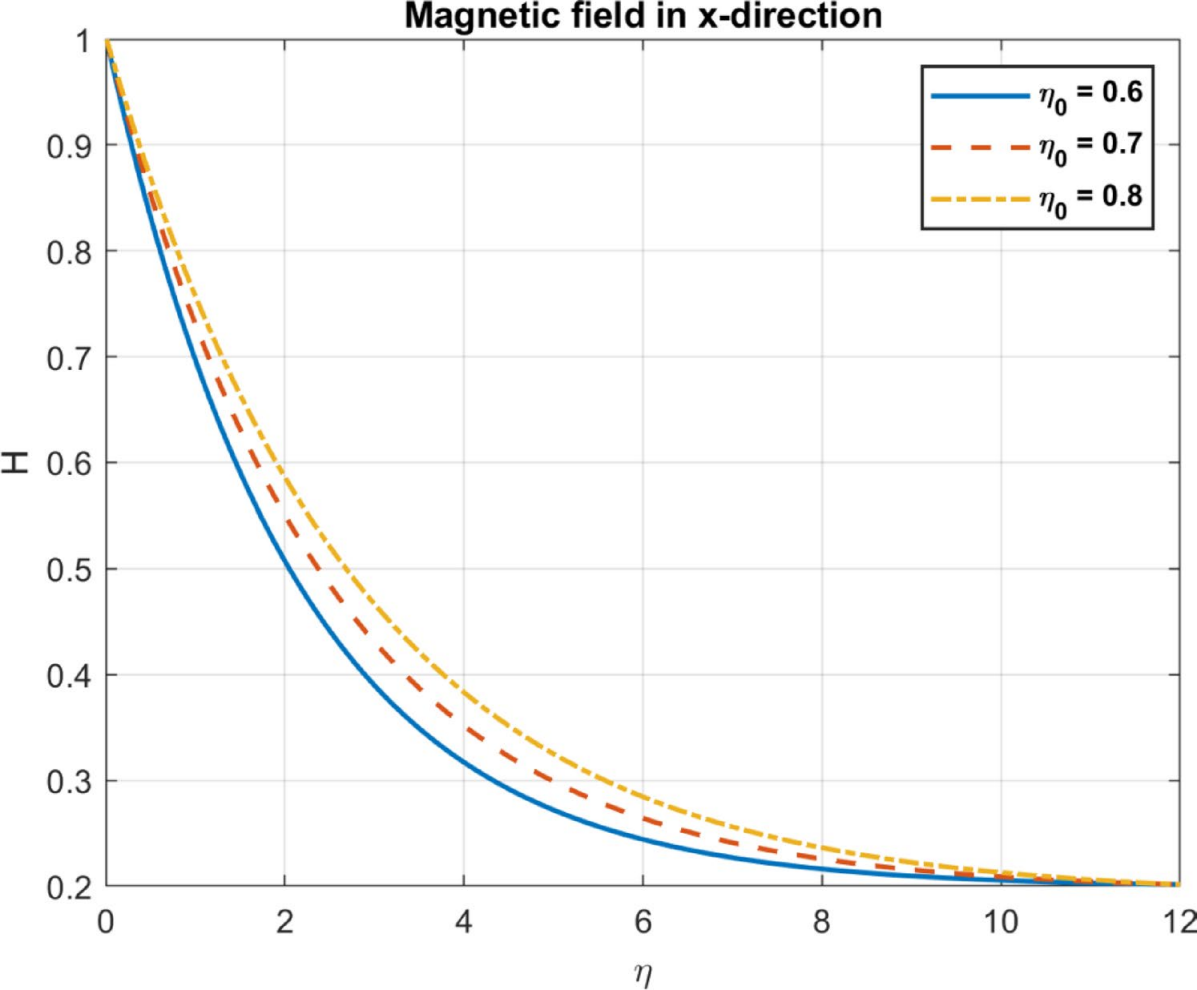
The influence of *η*_*0*_ on magnetic field in x-direction.

### Chemical reaction rate parameter ($\:{\lambda\:}_{c}=\:{\text{k}}_{r}/\:{\text{D}}_{r}$)

The relationship between the density of the reacting species and the chemical reaction rate parameter may be understood via the use of diffusion processes and chemical reaction dynamics. In a system where both reaction and diffusion are occurring, if the reaction rate increases without the diffusion rate increasing simultaneously, the reactants will be depleted faster than they can permeate into the reaction zone. This imbalance leads to a decrease in the local density of the responsive species. These results are shown in Fig. 10.

### Nonlinear thermophoresis parameter ($\:{S}_{t}=\:{\text{D}}_{T}\:/\:\left({\text{T}}_{\infty\:}\text{*}{\text{D}}_{B}\right)$)

Thermophoresis refers to the movement of particles in a fluid due to temperature gradients. In some cases, thermophoretic behavior may become nonlinear under strong temperature gradients or complex interactions. The effect on different phenomena including temperature, heat flux, and velocity can be described as follows:

As the Nonlinear Thermophoresis Parameter is increased, temperature gradients tend to produce zones of lower temperature near the particles. Thermophoresis redistributes particles, affecting local temperature fields due to altered thermal conductivity. A reduction in temperature gradients leads to decreased heat flux. Because temperature gradients largely drive heat flux, in some cases, thermophoresis may reduce effective thermal gradients, altering local heat flux. Figures 11, 12 and 13 show these results.

### Hartmann number $\:\left(M\:=\:\left(\sigma\:\:{B}_{0}^{2}\:L\right)\:/\:{\mu\:}_{hnf}\right)$

In ferrohydrodynamics, the Hartmann number (M) quantifies the influence of a magnetic field on electrically conducting fluid flow. Its influence on reducing velocity can be stated as follows:

The fluid’s velocity drops when magnetic forces outweigh viscous forces. Greater magnetic dampening is indicated by a higher Hartmann number. Lorentz forces, resulting from the interaction between electrical currents in the fluid and magnetic fields, resist fluid motion. Stronger magnetic fields can thicken the boundary layer by damping fluid motion. In Fig. 14, they are depicted.

### Bioconvection parameter (Péclet number), $\:Pe$

The bioconvection parameter reflects the impact of microbial motility on flow and is influenced by but distinct from the Péclet number, which represents the ratio of advective to diffusive transport, but incorporates additional factors specific to bioconvection. As $\:Pe$ increases, it influences the bacterial density and density gradients in the following ways. As the bioconvection parameter increases, microbial-induced advective transport, influenced by the Péclet number ( $\:Pe$), prevails over diffusion, Enhanced advection can redistribute microbial populations, potentially reducing localized density gradients. Redistributes bacterial density, decreasing local concentrations in high-density regions. Strong advection forces the bacteria out of constricted regions, distributing them more evenly in the fluid. As a result, bacterial concentrations are less dense, reducing the overall density gradient. An increased bioconvection parameter promotes uniform bacterial distribution by enhancing advective transport, reducing density gradients, as shown in Figs. 15 and 16.

**Fig. 25:**
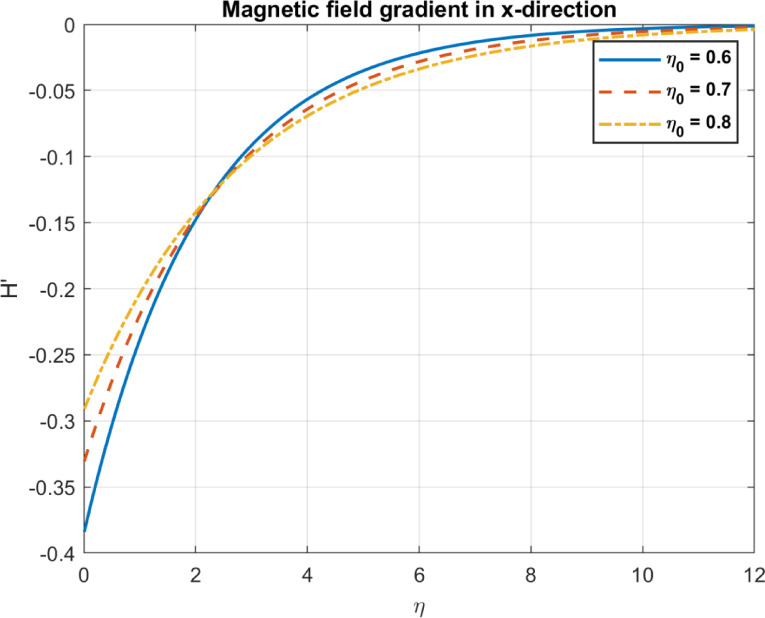
The influence of *η*_*0*_ on magnetic field gradient in x-direction.

### Electric conductivity ratio $\:(\varLambda\:=\:{{\upmu\:}}_{e}\:/\:{{\upmu\:}}_{hnf})$

The electric conductivity ratio is the ratio of the electrical conductivity of the hybrid nanofluid to that of the base fluid. Its effects on velocity reduction can be explained as follows:

As the electrical conductivity ratio increases, higher electrical conductivity enhances Lorentz forces, which resist fluid motion and reduce the velocity profile. This effect is due to the enhanced Lorentz forces, which increase resistance to flow, thicken the momentum boundary layer, and suppress fluid motion. Higher $\:\varLambda\:$ ​ enhances Lorentz forces, Lorentz forces suppress fluid motion, reducing the velocity gradient near walls and potentially thinning the boundary layer. as shown in Fig. 17.

### Pollutant decay rate $\:\left(\alpha\:\right)$

The pollutant decay rate, typically denoted by $\:\alpha\:$, represents the rate of exponential decrease in contaminant concentration. As $\:\alpha\:$ increases, pollutants dissipate more rapidly, leading to a faster reduction in their concentration within the fluid. Lower concentrations of pollutants reduce their influence on system dynamics, potentially aiding remediation by enhancing bioconvection efficiency through improved microbial motion and fluid mixing. A higher pollutant decay rate ($\:\alpha\:$) accelerates the exponential reduction in contaminant concentration, aiding fluid remediation. as shown in Figs. 18 and 19.

### Diffusion coefficient for pollutants $\:Dp$

The diffusion coefficient of pollutants dictates how the pollutants would behave and spread in different environments and therefore has an active role in influencing pressure dynamics directly. More diffusive pollutants with a larger diffusion coefficient spread out more quickly, changing concentration gradients and nearby pollutant concentrations. Larger concentration gradients lead to more rapid diffusion as the system approaches equilibrium, causing temporary pressure variations. These effects are illustrated in Fig. 20.

**Fig. 26:**
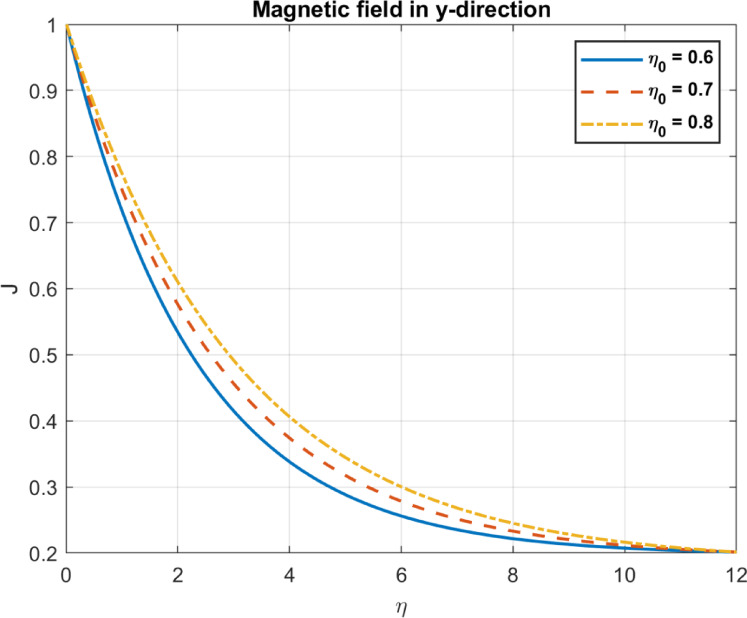
The influence of *η*_*0*_ on magnetic field in y-direction

### Diffusion coefficient for the reacting species $\:{D}_{r}$

As they undergo chemical reactions, the reacting species’ diffusion coefficient plays a decisive role in determining their behavior and density. A higher diffusion coefficient allows reacting species to diffuse more quickly through the medium, leading to a more uniform concentration and density distribution. With the rise of reactive species concentration, their density also rises. A higher diffusion coefficient facilitates better mixing, leading to better uniform density in the reaction medium. These are shown in Fig. 21.

### Diffusion coefficient for the microorganisms $\:{D}_{n}$

Microorganisms, such as bacteria, have a diffusion coefficient that plays a significant role in determining their concentration and density gradients. As the diffusion coefficient increases, the bacteria move rapidly in the nanofluid producing a more evenly distributed density. As bacteria distribute themselves, some areas become denser, particularly where nutrient levels or reproduction is high. Brisk diffusion ensures relatively stable levels of density, however, irrespective of changes around them. The slope of the concentration gradients is influenced by the diffusion coefficient. These effects are illustrated in Figs. 22 and 23.

### Magnetic diffusivity, $\:{\eta\:}_{0}$

**Fig. 27:**
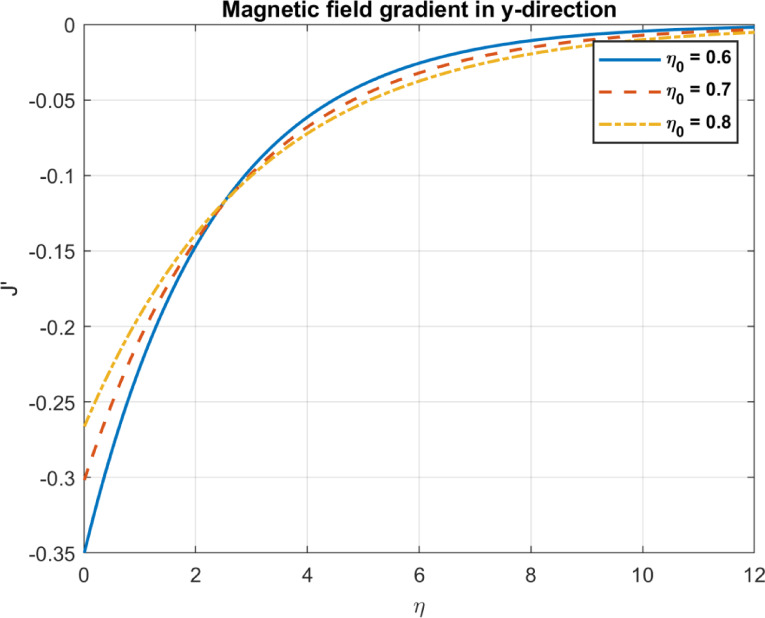
The influence of $\:{\eta\:}_{0}$ on magnetic field gradient in y-direction.

A magnetic field’s rate of diffusion into a medium is measured by its magnetic diffusivity. A material with a higher magnetic diffusivity may be penetrated by the magnetic field more quickly and deeply, which produces a more even distribution across the medium. High magnetic diffusivity materials react to changes in the external magnetic field sooner. Therefore, the internal magnetic field changes more quickly in response to any change in the external field. Figures 24, 25, 26 and 27 provide examples of these impacts.

### Numerical implementation and visualization

The system of transformed ordinary differential equations (Eqs. 34–42) was solved numerically using MATLAB’s bvp4c solver, a finite difference method tailored for boundary value problems. We employ adaptive step-size control to ensure accuracy, with an initial grid in the similarity variable generated using MATLAB’s linspace function and refined adaptively by bvp4c. Step sizes of $\:h\:=\:0.1\:,\:0.05\:,\:0.025$ were tested against a reference step size of $\:{h\:}_{ref}\:=\:0.001$ to verify convergence (Tables 2 and 3). we iterate to satisfy the boundary conditions at $\:\eta\:\:=\:0$ and $\:\eta\:\:\to\:\:\infty\:$ (Eq. 43), with a relative tolerance of$\:\:{10}^{-3}$. Parametric line plots (Figs. 5, 6, 7, 8, 9, 10, 11, 12, 13, 14, 15, 16, 17, 18, 19, 20, 21, 22, 23, 24, 25, 26 and 27), illustrating variables such as velocity, temperature, nanoparticle concentration, microbial density, and pollutant concentration, were generated using MATLAB’s plot function. Contour plots were not utilized in this study, as the results focus on one-dimensional profiles as functions of . If contour plots were required, MATLAB’s contour function would be employed. The implementation in MATLAB ensures robust numerical solutions and high-quality visualizations, with results validated against Rashed et al.^57^ (Fig. 2).

## Conclusion

This paper provides to a comprehensive parametric analysis of coupled bioconvection processes in combination with magnetized fluids, focusing on the remediation of nanoparticles and pollutant transport for sustainable water treatment. The study declares the importance of bioconvection on the dispersion and removal of pollutants, particularly through the magnetized fluids. The combination of nanoparticles, microorganisms, and a magnetic field is the reason for the efficient, dynamic transport of pollutants, a technique that brings about a completely novel water purification approach. Other parameters such as Brownian motion, thermophoresis, Hartmann number, and diffusion coefficients will have important roles to play in the regulation of heat transfer, velocity distribution, as well as the migration of microbes in the fluid system. These results are a concrete example of the capacity of magnetic bioconvection to be tapped into for an enhanced level of control and efficacy of nanoparticle-mediated pollutant removal, an innovation that is introducing new ways of eco-friendly water treatment technologies.

Key Findings:

- Higher $\:{D}_{B}$ increases particle dispersion, reducing local energy transfer efficiency, leading to lower heat flux and localized cooling. It also enhances particle collision frequency, increasing mean velocity.
- Increased $\:{R}_{d}$ enhances radiative energy dissipation, lowering equilibrium temperature and reducing heat flux, as energy is emitted rather than stored or conducted.
- Higher $\:{\lambda\:}_{c}$ accelerates reactant consumption, outpacing diffusion, which reduces the local density of reacting species due to insufficient replenishment.
- Increased $\:{S}_{t}$ induces local cooling by absorbing thermal energy, reducing temperature and heat flux. It also influences velocity by altering particle motion dynamics.
- Higher $\:\:M$increases Lorentz forces, damping fluid flow, reducing velocity, and thinning the boundary layer, which enhances control over pollutant movement.
- Higher $\:\:Pe$promotes advective transport, spreading bacteria more uniformly, reducing bacterial density and density gradients, and enhancing pollutant dispersion.
- Higher $\:\:\varLambda\:$strengthens electromagnetic damping and viscosity, reducing fluid velocity by increasing resistance to flow.
- $\:\alpha\:$ represents pollutant pressure decay. Lower pollutant concentrations reduce partial pressure (per Raoult’s law), enhancing nanoparticle-driven pollutant removal and improving water purification efficiency.
- Higher $\:{D}_{p}$ accelerates diffusion, reducing concentration gradients and causing temporary pressure variations as the system approaches equilibrium, aiding pollutant dispersion.
- Higher $\:{D}_{r}$ promotes uniform concentration and density distribution, improving mixing and increasing species density in reaction zones.
- Higher $\:{D}_{n}$ enhances bacterial dispersion, stabilizing density distribution and reducing concentration gradients, supporting consistent microbial activity in pollutant removal.
- Higher $\:{\eta\:}_{0}$ enables faster and deeper magnetic field penetration, ensuring uniform distribution and rapid response to external field changes, enhancing fluid control.

Researchers make substantial attempts to examine the application’s boundaries. In the future, significant study will be undertaken on hybrid nanofluids that increase magnetic field and integration with biological systems. The system’s architecture may allow for innovation. The increased magnetic field in microchannels allows contaminants to be removed more quickly. To combat those particularly persistent organic contaminants, we may explore a strong collaboration: adding advanced oxidation mechanisms directly into the bioconvection architecture, resulting in a multifaceted attack on water pollution. Researchers make substantial attempts to examine the application’s boundaries. In the future, significant study will be undertaken on hybrid nanofluids that increase magnetic field and integration with biological systems.

## Data Availability

All data generated or analysed during this study are included in this published article.

## Latin symbols

${\text{A}}_{1},,,{\text{A}}_{24}$: Coefficients appearing in the transformation of the ODE system
${\text{B}}_{0}$: Magnetic field strength
$\text{C}$: Concentration of nanoparticles
${\text{C}}_{\text{w}}$: Concentration at wall
$${\text{C}}_{\infty }$$: Ambient concentration
${\text{C}}^{*}$: Dimensionless form of concentration
$${\text{c}}_{1},{\text{c}}_{2},{\text{c}}_{3},,,,$$: Constants
${\text{D}}_{\text{B}}$: Brownian motion coefficient
${\text{D}}_{\text{T}}$: Thermal diffusion coefficient
${\text{D}}_{\text{r}}$: Diffusion coefficient for the reacting species
${\text{D}}_{\text{n}}$: Diffusion coefficient for the microorganisms
${\text{D}}_{\text{p}}$: Diffusion coefficient for pollutants
$${\text{g}}_{1}$$: Gravitational acceleration
$$\text{G}$$: Group structure
${\text{H}}_{1}$: Intensity of magnetic field in x-direction
${\text{H}}_{2}$: Intensity of magnetic field in y-direction
${{\text{H}}_{1}}_{\text{w}},{{\text{H}}_{2}}_{\text{w}}$: Stretching magnetic flux
${\text{H}}_{1}^{*},{\text{H}}_{2}^{*}$: Dimensionless forms of magnetic field components
${\text{K}}^{\text{s}},{\text{Q}}^{\text{s}}$: Differential coefficient functions
${\text{k}}_{\text{r}}$: Rate constant for the chemical reaction
${\text{k}}_{\text{hnf}}$: Thermal conductivity of hybrid nanofluid
$$\text{N}$$: Microorganisms’ concentration
${\text{N}}_{\text{w}}$: Microorganisms at wall
$${\text{N}}_{\infty }$$: Ambient microorganisms
${\text{N}}^{*}$: Dimensionless form of microorganisms
$$\text{M}$$: Hartmann number
$\text{P}$: Concentration of pollutants in the fluid
${\text{P}}^{*}$: Dimensionless form of pollutant concentration
$${\text{P}}_{\text{e}}$$: Péclet number
$\text{Q}$: Heat generation term
$\text{R}$: Concentration of the reacting species
${\text{R}}^{*}$: Dimensionless form of reacting specie concentration
$$\text{S}$$: System variables
${\text{S}}_{\text{i}}$: Original dependent variables of the system
${\text{S}}_{\text{t}}$: Nonlinear thermophoresis parameter
$\text{T}$: Temperature of nanoparticles
${\text{T}}_{\text{w}}$: Temperature at wall
$${\text{T}}_{\infty }$$: Ambient temperature
$\Delta \text{T},\Delta \text{C},\Delta \text{N}$: Temperature, concentration, and microorganism concentration differences
$\text{u}$: Velocity component in x-direction
${\text{u}}^{*}$: Dimensionless form of velocity
$${\text{U}}_{\text{w}},{\text{V}}_{\text{w}}$$: Stretching velocity
$\text{v}$: Velocity component in y-direction
$$\text{x},\text{y},\text{t}$$: Independent variables

## Greek symbols

$\upeta$: A transformed variable related to the spatial coordinate and time
$${\upeta }_{0}$$: Magnetic diffusivity
${\uprho }_{\text{p}}$: Density of the particles
${\uprho }_{\text{f}}$: Density of the fluid
${\uprho }_{\text{m}}$: Density of microorganisms
${\upmu }_{\text{hnf}}$: Dynamic viscosity of the hybrid nanofluid
${\uprho }_{\text{hn}}$: Density of the hybrid nanofluid
$\upbeta$: Coefficient representing the thermal expansion
$$\upsigma$$: Magnetic susceptibility
$\alpha$: Coefficient related to the rate of pollutant decay
$\upgamma$: Coefficient representing the influence of temperature on pollutant dispersion
${\upzeta }^{*}$: Coefficient indicating the effect of concentration on pollutant behavior
$\updelta$: Coefficient representing the influence of the magnetic field on pollutant dispersion
${\left(\uprho {\text{c}}_{\text{p}}\right)}_{\text{hnf}}$: Volumetric heat capacity of hybrid nanofluid
$${\upmu }_{\text{e}}$$: Magnetic permeability
$\uptheta$: Dimensionless form of temperature
$\alpha _{1} ,\alpha _{2}$: Continuous symmetry parameters for group transformations
${\uplambda }_{\text{c}}$: Chemical reaction rate parameter

## References

[1] Waqas, H. et al. Numerical simulation for bioconvectional flow of burger nanofluid with effects of activation energy and exponential heat source/sink over an inclined wall under the swimming microorganisms. *Sci. Rep.* **11** (1), 14305 (2021). 34253797 10.1038/s41598-021-93748-x PMC8275614

[2] Alharbi, F. M. et al. Bioconvection due to gyrotactic microorganisms in couple stress hybrid nanofluid laminar mixed convection incompressible flow with magnetic nanoparticles and chemical reaction as carrier for targeted drug delivery through porous stretching sheet. *Molecules* **26**10.3390/molecules26133954 (2021). 10.3390/molecules26133954 PMC8271748 34203543

[3] Shi, Q. H. et al. Numerical study of bio-convection flow of magneto-cross nanofluid containing gyrotactic microorganisms with activation energy. *Sci. Rep.* **11** (1), 16030 (2021). 34362971 10.1038/s41598-021-95587-2 PMC8346499

[4] Rashed, A. S., Mahmoud, T. A. & Kassem, M. M. Behavior of nanofluid with variable brownian and thermal‎ diffusion coefficients adjacent to a moving vertical plate. *J. Appl. Comput. Mech.* **7** (3), 1466–1479 (2021).

[5] Rashed, A., Mahmoud, T. & Kassem, M. Analysis of homogeneous steady state nanofluid surrounding cylindrical solid pipes. *Egypt. Int. J. Eng. Sci. Technol.* **31** 71–82. (2020).

[6] Mabrouk, S. M. et al. Influence of power-law index and hybrid-nanoparticles concentrations on the behavior of non-Newtonian hybrid nanofluid inside water solar collector. *Mod. Phys. Lett. B*. **38** (05), 2350226 (2023).

[7] Mabrouk, S. et al. Thermal and entropy behavior of sustainable solar energy in water solar collector due to non-Newtonian power-law hybrid nanofluid. *Front. Energy Res.* **11**. (2023).

[8] Waqas, H. et al. Falkner-Skan time-dependent bioconvrction flow of cross nanofluid with nonlinear thermal radiation, activation energy and melting process. *Int. Commun. Heat Mass Transfer*. **120**, 105028 (2021).

[9] Rashed, A. S. Analysis of (3 + 1)-dimensional unsteady gas flow using optimal system of lie symmetries. *Math. Comput. Simul.* **156**, 327–346 (2019).

[10] Rashed, A. S., Nasr, E. H. & Kassem, M. M. Similarity analysis of mass and heat transfer of FHD steady flow of nanofluid incorporating magnetite nanoparticles (Fe3O4). (2020).

[11] Rashed, A. S., Mabrouk, S. M. & Wazwaz, A. M. Unsteady three-dimensional laminar flow over a submerged plate in electrically conducting fluid with applied magnetic field. *Waves Random Complex. Media*. **33** (3), 505–524 (2023).

[12] Rashed, A. S., Mabrouk, S. M. & Wazwaz, A. M. Forward scattering for non-linear wave propagation in (3 + 1)-dimensional Jimbo-Miwa equation using singular manifold and group transformation methods. *Waves Random Complex. Media*. **32** (2), 663–675 (2022).

[13] Saleh, R., Rashed, A. S. & Wazwaz, A. M. Plasma-waves evolution and propagation modeled by sixth order Ramani and coupled Ramani equations using symmetry methods. *Phys. Scr.* **96** (8), 085213 (2021).

[14] Sarma, N. & Paul, A. Thermophoresis and Brownian motion influenced bioconvective cylindrical shaped Ag–CuO/H2O Ellis hybrid nanofluid flow along a radiative stretched tube with inclined magnetic field. *BioNanoScience* (2023).

[15] Paul, A., Sarma, N. & Patgiri, B. Mixed convection of shear-thinning hybrid nanofluid flow across a radiative unsteady cone with Suction and slip effect. *Mater. Today Commun.* **37**, 107522 (2023).

[16] Rafique, K. et al. Investigation of thermal stratification with velocity slip and variable viscosity on MHD flow of Al2O3 – Cu – TiO2/H2O nanofluid over disk. *Case Stud. Therm. Eng.* **49**, 103292 (2023).

[17] Paul, A., Sarma, N. & Patgiri, B. Thermal and mass transfer analysis of Casson-Maxwell hybrid nanofluids through an unsteady horizontal cylinder with variable thermal conductivity and Arrhenius activation energy. *Numer. Heat Trans. Part A Appl.* 1–26 (2023).

[18] Mahmood, Z. et al. Numerical analysis of MHD tri-hybrid nanofluid over a nonlinear stretching/shrinking sheet with heat generation/absorption and slip conditions. *Alexandria Eng. J.* **76**, 799–819 (2023).

[19] Islam, S. et al. Electroosmotic flow in ternary (TiO2-SiO2-Al2O3) blood-based sutterby nanomaterials with bio-active mixers. *Int. J. Thermofluids*. **18**, 100363 (2023).

[20] Islam, S. et al. Dynamics of chemically reactive Carreau nanomaterial flow along a stretching Riga plate with active bio-mixers and arrhenius catalysts. *Heliyon* **9** (11), e21727 (2023). 37954265 10.1016/j.heliyon.2023.e21727 PMC10637908

[21] Rana, B. et al. Swimming of microbes in entropy optimized nano-bioconvective flow of Prandtl–Erying fluid. *Heat. Transf.* **51**, pna–na (2022).

[22] Rana, B. M. J. et al. Swimming of microbes in blood flow of nano-bioconvective williamson fluid. *Therm. Sci. Eng. Progress*. **25**, 101018 (2021).

[23] Islam, M. S., Islam, S. & Siddiki, M. N. A. A. Numerical simulation with sensitivity analysis of MHD natural convection using Cu-TiO2-H2O hybrid nanofluids. *Int. J. Thermofluids*. **20**, 100509 (2023).

[24] Smrity, A. M. A. & Yin, P. Design and performance evaluation of pulsating heat pipe using metallic nanoparticles based hybrid nanofluids. *Int. J. Heat Mass Transf.* **218**, 124773 (2024).

[25] Bone, S. E., Steinrück, H. G. & Toney, M. F. Advanced characterization in clean water technologies. *Joule* **4** (8), 1637–1659 (2020).

[26] Van Vliet, M. T. et al. Global water scarcity including surface water quality and expansions of clean water technologies. *Environ. Res. Lett.* **16** (2), 024020 (2021).

[27] Macedonio, F. et al. Efficient technologies for worldwide clean water supply. *Chem. Eng. Process.* **51**, 2–17 (2012).

[28] Nagar, A. & Pradeep, T. Clean water through nanotechnology: needs, gaps, and fulfillment. *ACS Nano*. **14** (6), 6420–6435 (2020). 32433866 10.1021/acsnano.9b01730

[29] Acharya, N. Effects of the curved fins on the entropy analysis and hydrothermal variations of Buoyancy-driven MWCNT-Fe3O4-H2O hybrid nanofluid flow within an annular enclosure. *Appl. Therm. Eng.* **269**, 126100 (2025).

[30] Acharya, N. Framing the effect of fitted curved fins’ curvature on the flow patterns and entropy analysis of buoyancy-driven magnetized hybrid nanofluidic transport within an annular enclosure. *J. Energy Storage*. **100**, 113638 (2024).

[31] Acharya, N. Spectral simulation on the flow patterns and thermal control of radiative nanofluid spraying on an inclined revolving disk considering the effect of nanoparticle diameter and solid–liquid interfacial layer. *J. Heat Transfer*. **144** (9), 092801 (2022).

[32] Acharya, N. On the flow patterns and thermal behaviour of hybrid nanofluid flow inside a microchannel in presence of radiative solar energy. *J. Therm. Anal. Calorim.* **141** (4), 1425–1442 (2020).

[33] Acharya, N. Spectral quasi linearization simulation on the hydrothermal behavior of hybrid nanofluid spraying on an inclined spinning disk. *Partial Differ. Equations Appl. Math.* **4**, 100094 (2021).

[34] Adhikari, R. & Das, S. Exploring microbial dynamics in a reactive magnetised Casson-Maxwell-Oldroyd-B nanofluid on a slanted elongated cylinder: entropy assessment. *Int. J. Ambient Energy*. **45** (1), 2367743 (2024).

[35] Sarkar, S. & Das, S. Dynamics of oxytactic microbes-infused cross nanofluid around a stretchy cylinder subject to Lorentz force, arrhenius activation energy, and nonlinear thermal radiation. *Eur. Phys. J. Plus*. **139** (2), 120 (2024).

[36] Adhikari, R. & Das, S. Biological transmission in a magnetized reactive Casson–Maxwell nanofluid over a Tilted stretchy cylinder in an entropy framework. *Chin. J. Phys.* **86**, 194–226 (2023).

[37] Sarkar, S. & Das, S. Gyrotactic microorganisms swimming in magneto-Sutterby-nanofluid over a sliding cylinder set in a Darcy-Forchheimer porous space with arrhenius kinetics. *Int. J. Ambient Energy*. **45** (1), 2258896 (2024).

[38] Sarkar, S. & Das, S. Gyrotactic microbes’ movement in a magneto-nano-polymer induced by a stretchable cylindrical surface set in a DF porous medium subject to non-linear radiation and arrhenius kinetics. *Int. J. Model. Simul.* 1–18. (2023).

[39] Ali, A., Sarkar, S. & Das, S. Bioconvective chemically reactive entropy optimized Cross-nano-material conveying oxytactic microorganisms over a flexible cylinder with Lorentz force and arrhenius kinetics. *Math. Comput. Simul.* **205**, 1029–1051 (2023).

[40] Animasaun, I. L. et al. *Ratio of Momentum Diffusivity To Thermal Diffusivity: Introduction, meta-analysis, and Scrutinization* (Chapman and Hall/CRC, 2022).

[41] Wang, F. et al. Dynamics through three-inlets of t-shaped ducts: significance of Inlet velocity on transient air and water experiencing cold fronts subject to turbulence. *Int. Commun. Heat Mass Transfer*. **148**, 107034 (2023).

[42] Animasaun, I., Muhammad, T. & Yook, S. J. Exploration of half‐cycle length of converging circular wavy duct with diverging‐outlet: Turbulent water dynamics. *Adv. Theory Simul.* 2500038. (2025).

[43] Zhu, H., Chen, S. & Zhang, H. Performance prediction and manipulation strategy of a hybrid system based on tubular solid oxide fuel cell and annular thermoelectric generator. *J. Non-Equilib. Thermodyn.* **50** (1), 149–172 (2025).

[44] Cantrell, R. S. & Cosner, C. *Spatial Ecology Via reaction-diffusion Equations* (J. Wiley, 2003).

[45] Becker, S. & Kuznetsov, A. V. *Transport in Biological Media* (Elsevier/Academic, 2013).

[46] Ming, T. et al. *Pollutant Dispersion in Built Environment* (Springer, 2017).

[47] Cantrell, R. S. & Cosner, C. *Spatial Ecology Via reaction-diffusion Equations* (Wiley, 2004).

[48] Rashed, A. S., Nasr, E. H. & Kassem, M. M. Mathematical investigation for flow characteristics of laminar ferro-nanofluid incorporating Cobalt ferrite nanoparticles. *J. Nano Res.* **68**, 52–69 (2021).

[49] Ming, T. et al. *Pollutant Dispersion in Built Environment* (Springer, 2017).

[50] Rashed, A. S., Nasr, E. H. & Kassem, M. M. Boundary layer analysis adjacent to moving heated plate inside electrically conducting fluid with heat source/sink. *Int. J. Heat Technol.* **38** (3), 682–688 (2020).

[51] Rashed, A. S., Nasr, E. H. & Kassem, M. M. Similarity analysis of mass and heat transfer of FHD steady flow of nanofluid incorporating magnetite nanoparticles (Fe_*3*_*O*_*4*_) East African Scholars. *J. Eng. Comput. Sci.* **3** (4), 54–63 (2020).

[52] Mabrouk, S. & Rashed, A. Analysis of (3 + 1)-dimensional Boiti–Leon–Manna–Pempinelli equation via lax pair investigation and group transformation method. *Comput. Math Appl.* **74** (10), 2546–2556 (2017).

[53] Saleh, R., Kassem, M. & Mabrouk, S. Exact solutions of Calgero–Bogoyavlenskii–Schiff equation using the singular manifold method after Lie reductions. *Math. Methods Appl. Sci.* **40** (16), 5851–5862. (2017).

[54] Rashed, A. & Kassem, M. Group analysis for natural convection from a vertical plate. *J. Comput. Appl. Math.* **222** (2), 392–403 (2008).

[55] Mabrouk, S. & Kassem, M. Group similarity solutions of (2 + 1) Boiti-Leon-Manna-Pempinelli lax pair. *Ain Shams Eng. J.* **5** (1), 227–235 (2014).

[56] Mabrouk, S., Kassem, M. & Abd-el-Malek, M. Group similarity solutions of the lax pair for a generalized Hirota–Satsuma equation. *Appl. Math. Comput.* **219** (14), 7882–7890 (2013).

[57] Rashed, A. S., Nasr, E. H. & Mabrouk, S. M. Influence of gyrotactic microorganisms on bioconvection in electromagnetohydrodynamic hybrid nanofluid through a permeable sheet. *Computation* **12** (1), 17 (2024).

[58] Pedley, T. J., Hill, N. & Kessler, J. O. The growth of bioconvection patterns in a uniform suspension of gyrotactic micro-organisms. *J. Fluid Mech.* **195**, 223–237 (1988). 11543357 10.1017/s0022112088002393

[59] Wen, D. et al. Review of nanofluids for heat transfer applications. *Particuology* **7** (2), 141–150 (2009).

[60] Haddad, Z. et al. Natural convection in nanofluids: are the thermophoresis and brownian motion effects significant in nanofluid heat transfer enhancement? *Int. J. Therm. Sci.* **57**, 152–162 (2012).

[61] Odenbach, S. Recent progress in magnetic fluid research. *J. Phys.: Condens. Matter*. **16** (32), R1135 (2004).

[62] Rosensweig, R. Directions in ferrohydrodynamics. *J. Appl. Phys.* **57** (8), 4259–4264 (1985).

[63] Yao, K. M., Habibian, M. T. & O’Melia, C. R. Water and waste water filtration. Concepts and applications. *Environ. Sci. Technol.* **5** (11), 1105–1112 (1971).

[64] Okubo, A. & Levin, S. A. *Diffusion and Ecological Problems: Modern Perspectives* **14** (Springer Science & Business Media, 2002).

[65] Li, L. et al. Insight into turbulent Reynolds number at the regular, converging, and diverging outlets: dynamics of air, water, and kerosene through y-shaped cylindrical copper ducts. *Int. Commun. Heat Mass Transfer*. **159**, 108044 (2024).

[66] Wang, F. et al. Transient cold-front-water through y-shaped aluminium ducts: nature of turbulence, non-equilibrium thermodynamics, and velocity at the converged and diverged outlets. *J. Non-Equilib. Thermodyn.* **49** (4), 485–512 (2024).

